# Induction of Endoplasmic Reticulum Stress-Mediated Apoptosis by Aminosteroid RM-581 Efficiently Blocks the Growth of PC-3 Cancer Cells and Tumors Resistant or Not to Docetaxel

**DOI:** 10.3390/ijms222011181

**Published:** 2021-10-17

**Authors:** René Maltais, Jenny Roy, Martin Perreault, Sachiko Sato, Julie-Christine Lévesque, Donald Poirier

**Affiliations:** 1 Laboratory of Medicinal Chemistry, Endocrinology and Nephrology Unit, CHU de Québec—Research Center, Québec, QC G1V 4G2, Canada; rene.maltais@crchudequebec.ulaval.ca (R.M.); jenny.roy@crchudequebec.ulaval.ca (J.R.); martin.perreault@crchudequebec.ulaval.ca (M.P.); 2 Bioimaging Platform, CHU de Québec—Research Center, Faculty of Medicine, Laval University, Québec, QC G1V 4G2, Canada; sachiko.sato@crchudequebec.ulaval.ca (S.S.); jc.levesque@crchudequebec.ulaval.ca (J.-C.L.); 3 Department of Molecular Medicine, Faculty of Medicine, Université Laval, Québec, QC G1V 0A6, Canada

**Keywords:** prostate cancer, aminosteroid, endoplasmic reticulum stress, cholesterol, fatty acid

## Abstract

Aminosteroid derivative RM-581 was previously identified as an endoplasmic-reticulum (ER) stress inducer with potent in vitro and in vivo anticancer activities. We report its evaluation in androgen-independent prostate cancer (PC-3) cells. RM-581 efficiently blocks PC-3 cell proliferation with stronger activity than that of a selection of known antineoplastic agents. This later also showed a synergistic effect with docetaxel, able to block the proliferation of docetaxel-resistant PC-3 cells and, contrary to docetaxel, did not induce cell resistance. RM-581 induced an increase in the expression level of ER stress-related markers of apoptosis, potentially triggered by the presence of RM-581 in the ER of PC-3 cells. These in vitro results were then successfully translated in vivo in a PC-3 xenograft tumor model in nude mice, showing superior blockade than that of docetaxel. RM-581 was also able to stop the progression of PC-3 cells when they had become resistant to docetaxel treatment. Concomitantly, we observed a decrease in gene markers of mevalonate and fatty acid pathways, and intratumoral levels of cholesterol by 19% and fatty acids by 22%. Overall, this work demonstrates the potential of an ER stress inducer as an anticancer agent for the treatment of prostate cancers that are refractory to commonly used chemotherapy treatments.

## 1. Introduction

Prostate cancer (PCa) is the most frequent malignancy in males, affecting one in seven men during their lifetime [1]. Important therapeutic innovations emerged in the last decade and significantly improved the global survival rate to PCa [2,3]. Despite this progress, the disease remains the second leading cause of death by cancer in North America [4]. A large percentage of deaths are due to the metastatic stage of PCa for which a limited number of valuable therapeutic options are available [5,6]. Typically, the first-line pharmacologic treatment of nonmetastatic PCa is based on androgen deprivation therapy (ADT), consisting in luteinizing hormones releasing agonists for chemical castration, with the possibility of combining this with antiandrogens for maximal androgen action blockade [7]. Unfortunately, patients who are initially androgen-sensitive are at risk of becoming resistant to ADT upon treatment, and subsequently develop a state named castration-resistant prostate cancer (CRPC) and progress toward metastasis [8,9].

Once CRPC occurs, chemotherapy usually takes the relay with docetaxel, a cytotoxic agent established in the last decade as the gold-standard therapy for resistant cancer cases [8]. However, the response duration to docetaxel remains quite limited, with an overall survival advantage of approximately three months [10]. Approximately half of CRPC patients treated with docetaxel do not initially respond, and the second half ultimately develop resistance to this antineoplastic agent [11]. This refractory character of CRPC toward docetaxel chemotherapy has mobilized research efforts to identify new therapeutic options. Abiraterone acetate (an inhibitor of androgen biosynthesis) [12], enzalutamide (a new generation of antiandrogen) [13], and cabazitaxel (a new generation of taxotere) [14] are three relevant examples of new drugs obtained from these research efforts that significantly impacted the median survival of CRPC patients [15,16]. However, resistance again occurs, even for those new drugs, leaving these resistant patients with no viable therapeutic options [17,18]. The development of innovative molecules acting by different mechanisms of action are thus urgently needed to give new hope to these doomed PCa patients [19].

Aminosteroid derivatives (AM) are a novel class of anticancer agents showing multipotent activity in vitro and in vivo in various cancer cell lines and tumors, including breast, pancreatic, and ovarian cancers [20,21,22,23]. AM leads to the disturbance of cholesterol homeostasis with the concomitant induction of endoplasmic reticulum (ER) stress that ultimately triggers apoptosis [23,24,25,26]. Among the AM family, RM-581 has emerged as the most valuable lead candidate [20,27,28]. Since ER stress inducers are a promising therapeutic approach for cancer treatment [29], including PCa [30], we assess RM-581’s potential for CRPC treatment. Used for over 30 years to study androgen-independent PCa and to evaluate the chemotherapeutic preclinical potential of drug candidates (in vitro and in vivo), we selected androgen-independent PC-3 human PCa cells as a valuable model of CRPC, since these cells are nonsensitive to androgens and are recognized to be one of the most aggressive PCa cell lines [31,32,33]. Considering the lack of novel therapeutics to treat advanced docetaxel-resistant PCa, we investigated the anticancer potency of RM-581 in PC-3 cells resistant or not to docetaxel, and in PC-3 xenograft tumors.

## 2. Results

### 2.1. RM-581 and RM-581-Fluo Antiproliferative Activities

**Figure 1:**
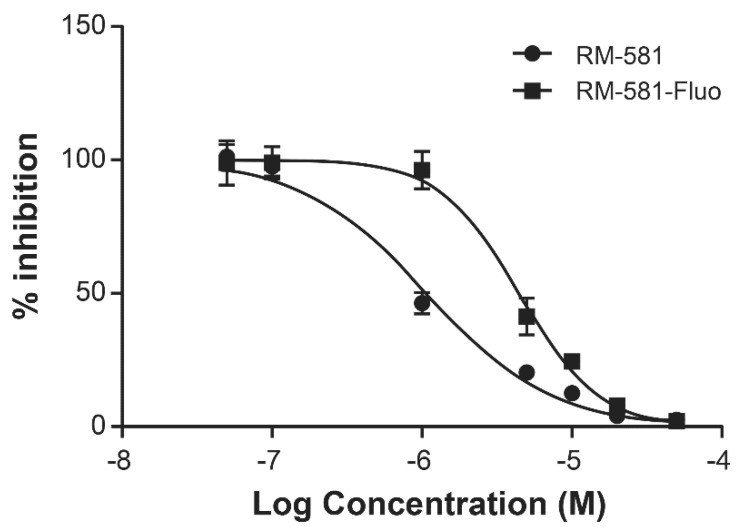
Comparison of effective concentrations reducing cell growth (EC_50_ values) of RM-581-Fluo vs. RM-581. PC-3 cells treated for 3 days with different concentrations of each aminosteroid. EC_50_ = 4.5 ± 0.3 and 1.1 ± 0.2 µM for RM-581-Fluo and RM-581, respectively. Mean of two experiments.

The antiproliferative potency of RM-581 was determined in androgen-dependent (LNCaP) and androgen-independent (DU-145 and PC-3) cell lines. RM-581 was active in the low micromolar range in those three PCa cell lines (LNCaP, DU-145, and PC-3) with EC_50_ of 1.2, 4.4 and 1.2 µM, respectively (Figure S1, Supplementary Materials). EC_50_ values of the fluorescent version of RM-581 (RM-581-Fluo) were also compared in PC-3 cells (1.1 and 4.5 µM, respectively) and were relatively close to each other (Figure 1). Considering their similar molecular structure, physicochemical and ADME properties (Tables S1 and S2, Supplementary Materials), and cytotoxic activity in PC-3 and MCF-7 cell lines, RM-581-Fluo is a valuable probe of RM-581 [34].

### 2.2. RM-581-Fluo Accumulates in ER of PC-3 Cells

**Figure 2:**
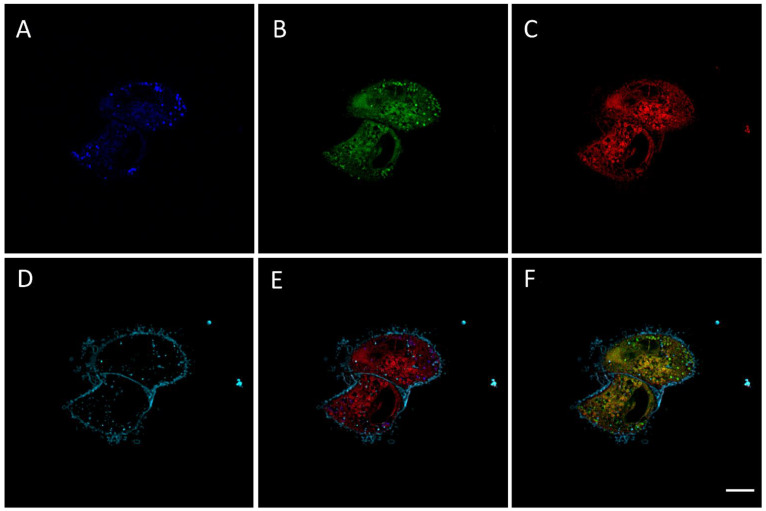
Confocal imaging of RM-581-Fluo in PC-3 cells. Representative image from confocal microscopy experiments in PC-3 cells at 60× magnification showing one focal plane. Cells treated for 2 h with 15 µM of RM-581-Fluo prior microscopy. (**A**) Cellular localization of RM-581-Fluo in blue (λex 405 nm, λem 460/50 nm); (**B**) cellular localization of RM-581-Fluo in green (λex 405 nm, λem 525/50 nm); (**C**) cellular localization of ER-Tracker in red (λex 561 nm, λem 593/40 nm); (**D**) cellular localization of Cell-mask in cyan (λex 642 nm, λem 700/75 nm); (**E**) merged images A, C, and D showing localization of RM-581-Fluo (blue), ER tracker (red) and Cell-mask (cyan); (**F**) merged images B, C, and D showing localization of RM-581-Fluo (green), ER tracker (red), Cell-mask (cyan), and colocalization of RM-581-Fluo and ER-Tracker (yellow). Scale bar: 10 µm. Three additional confocal images from a second experiment with RM-581-Fluo in PC-3 cells are shown in Figure S3, Supplementary Materials).

RM-581-Fluo is a fluorescent dye of RM-581 that was successfully used in MCF-7 cell imaging [34]. Having this unique tool in hand, we studied the distribution of RM-581 in PC-3 cells. The emission spectra of RM-581-Fluo (50 µM), which was excited at 405 nm, were first taken at three pH levels (7.5, 6.5, and 5.5) and did not show any difference, but when reducing the concentration to 30 and 5 µM, 2 emission peaks (460 and 525 nm) were observed (Figure S2, Supplementary Materials). Thus, confocal image acquisition was performed using 2 emission spectra at 460 and 525 nm. Confocal microscopy revealed that RM-581-Fluo was accumulated inside PC-3 cells treated with RM-581-Fluo for 2 h at 15 µM, suggesting the cell uptake of RM-581 (Figure 2). The majority of the RM-581-Fluo was proximal to the nucleus (Figure 2B), some of which appeared to be colocalized with ER-tracker (Figure 2F), while some RM-581-Fluo exhibited a punctuate structure, which was observed at both emissions (Figure 2A,B).

### 2.3. RM-581 Induces ER-Stress and Apoptosis

**Figure 3:**
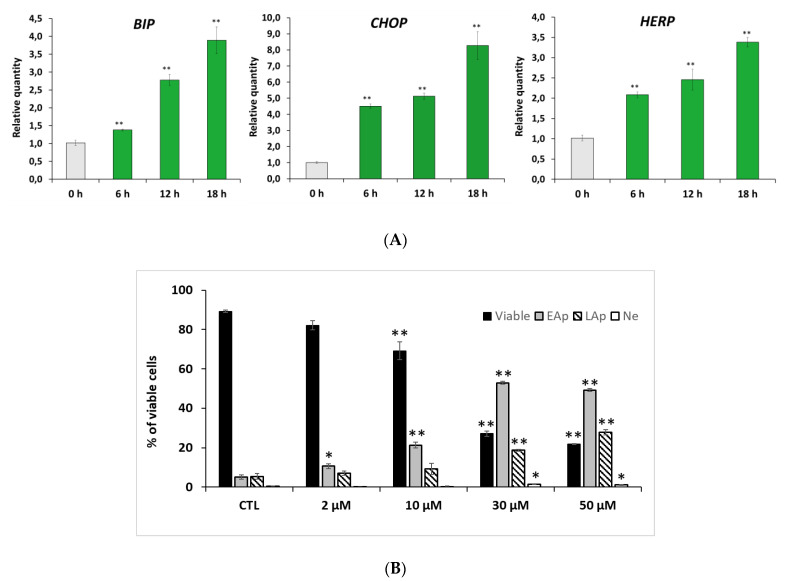
RM-581 triggers ER-stress mediated apoptosis of PC-3 cells. (**A**) mRNA expressions of *BIP*, *CHOP,* and *HERP* in PC-3 cells treated with RM-581 (5 µM) at 6, 12, and 18 h post-treatment. (**B**) Effect of RM-581 on viable, early apoptotic (EAp), late apoptotic (LAp), and necrotic (Ne) PC-3 cells. After cells (3 × 10^5^) had been exposed to RM-581 for 72 h at different concentrations, the number of each cell type was measured by flow cytometric analysis using propidium iodide dye. Each data point represents the mean of an experiment performed in triplicate (mean ± SD). * *p* < 0.05, ** *p* < 0.01 vs. 0 h or control (CTL).

PC-3 cells were treated in a time-course manner with RM-581 (5 µM), and the transcript levels of several markers of ER-stress related apoptosis (*BIP*, *CHOP,* and *HERP*) were analyzed by qPCR. RM-581 considerably raised the presence of *BIP*, *CHOP,* and *HERP* transcripts, which are the classical ER-stress apoptosis markers (Figure 3A) [35,36]. To assess the effect of RM-581 on PC-3 cell death, we proceeded with cell characterization by flow cytometry (Figure 3B). We observed a significant and increasing proportion of apoptotic cells (early and late) from 2 to 50 µM concentrations. At 30 and 50 µM, a major proportion of the cells entered the apoptotic stage. Consequently, viable cells dropped from 95% to 20% according to RM-581 concentration. No significant proportion of necrotic cells were observed at the different tested doses.

### 2.4. RM-581 Impairs Lipid Homeostasis

**Figure 4:**
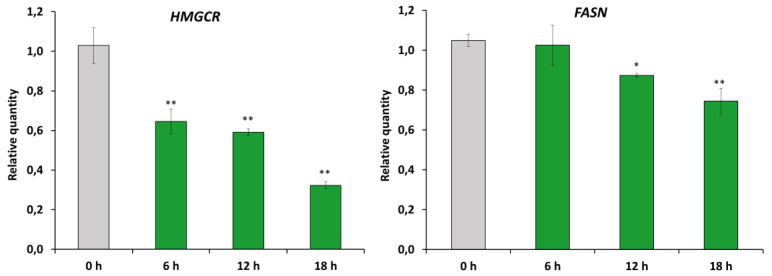
RM-581 affects lipid biosynthesis. mRNA expressions of *HMGCR* and *FASN* in PC-3 cells treated with RM-581 (5 µM) at 6, 12, and 18 h post-treatment. * *p* < 0.05, ** *p* < 0.01 vs. 0 h.

The effects of RM-581 (5 µM) on the expression of representative genes of the mevalonate and fatty acids (FA) biosynthesis pathways (*HMGCR* and *FASN*) were measured 6, 12, and 18 h after the treatment of PC-3 cells. A significant decrease in the mRNA expression of both genes was observed at the three time points for *HMGCR,* and at 12 and 18 h for *FASN* (Figure 4).

### 2.5. RM-581 Blocks the Growth of PC-3 Cells and Docetaxel-Resistant PC-3 Cells

**Figure 5:**
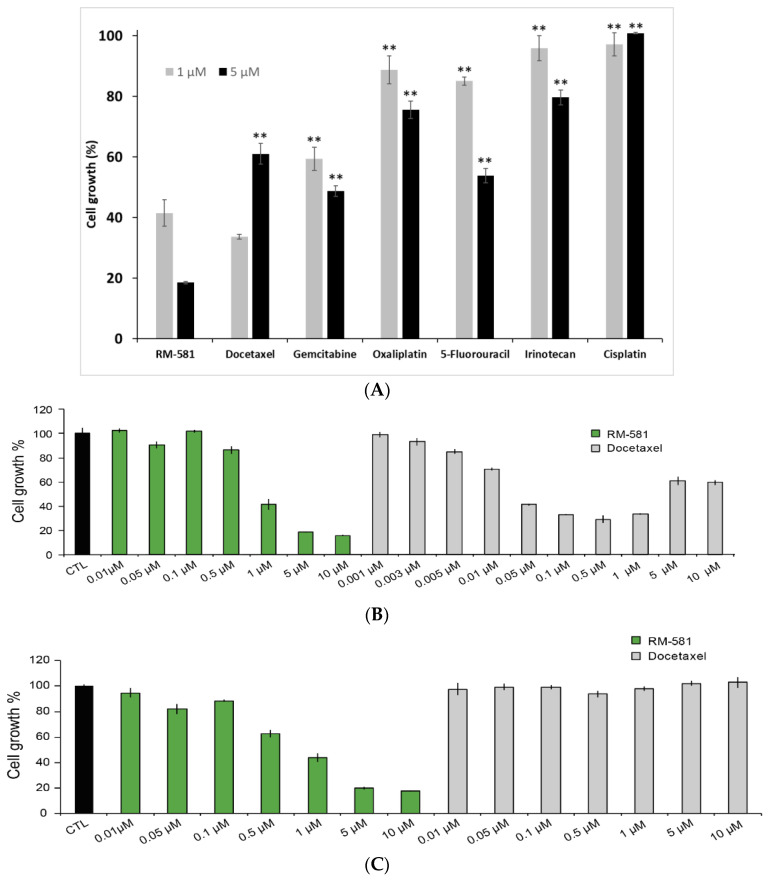

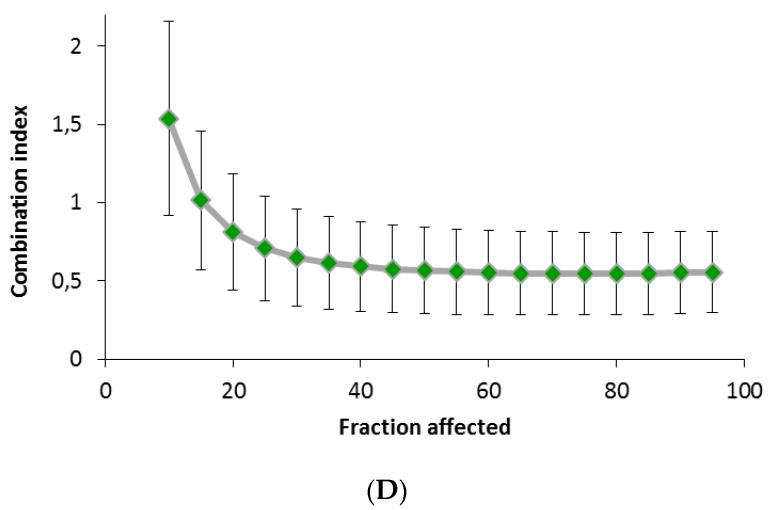
RM-581 blocks PC-3 cell proliferation. (**A**) Cytotoxic effects of RM-581 at 1 and 5 µM vs. a selection of current antineoplastic drugs. ** *p* < 0.01 vs. RM-581. (**B**) Effect of RM-581 and docetaxel on the proliferation of PC-3 cells at various concentrations (EC_50_ of RM-581 = 1.2 µM). (**C**) Effect of RM-581 and docetaxel on the proliferation of docetaxel-resistant PC-3 cells at various concentrations (EC_50_ of RM-581 = 0.9 µM). (**D**) RM-581 and docetaxel are synergistic anticancer agents in PC-3 cells. Combination index (CI) values were calculated using Chou–Talalay method [37]. Drug synergy, addition, and antagonism are defined by combination index (CI) values less than 1.0, equal to 1.0, or greater than 1.0, respectively. Two experiments were performed in triplicate ± SD.

The anticancer activity of RM-581 was first compared to a selection of six antineoplastic drugs used for the treatment of prostate cancer and other cancer types (Figure 5A). RM-581 was significantly more active than all the drugs tested at 1 and 5 µM except for docetaxel tested at 1 µM, which showed slight but significantly higher potency. As a second step, we performed an assay using multiple concentrations from 1 nM to 10 µM to better compare the anticancer potency between RM-581 and docetaxel (Figure 5B), a cytotoxic agent established in the last few decades as a gold standard for metastatic CRPC cases [8,10,11,17,18]. In this assay and others, the antiproliferative activity of docetaxel showed an unexpected profile depending on concentration. We observed a dose-response from 0.001 to 0.05 µM, a plateau from 0.05 to 1 µM, and lower cytotoxicity at higher concentrations of 5 and 10 µM.

The decrease in docetaxel efficiency in treating PC-3 cells suggests resistance. To verify this hypothesis, the potency of RM-581 was tested in docetaxel-resistant PC-3 cells (Figure 5C). After inducing drug resistance by treating the cells with docetaxel (10 µM) for 2 weeks, RM-581 showed the same antiproliferative effect (EC_50_ = 0.9 µM) as that previously obtained in PC-3 cells (EC_50_ = 1.2 µM, Figure 5B); docetaxel was inactive at all tested concentrations. Considering the limited anticancer action of docetaxel in PC-3 cells, and the appearance of resistance phenomena, we examined if RM-581, acting through a different mechanism of action than that of docetaxel, could have combination potential in PC-3 cells. The Chou–Talalay methodology revealed a synergistic effect of RM-581 and docetaxel with a CI value lower than 1 (Figure 5D).

PC-3 cells were also treated for several days with RM-581 at a concentration of 0.8 µM, and EC_50_ values were determined using the remaining cells after 12, 49, and 119 days of treatment (Figure S4, Supplementary Materials). Contrary to docetaxel that had induced cell resistance after only 12 days of treatment (Figure 5C), RM-581 did not induce resistance at the 3 tested treatment times of 12, 49, and 119 days (EC_50_ = 1.1, 1.6, and 0.8 µM, respectively). These 3 EC_50_ values were not significantly different from each other and when compared to PC-3 cells not treated with RM-581 (EC_50_ = 1.2 µM).

### 2.6. RM-581 Blocks Tumor Growth in PC-3 Xenografts

**Figure 6:**
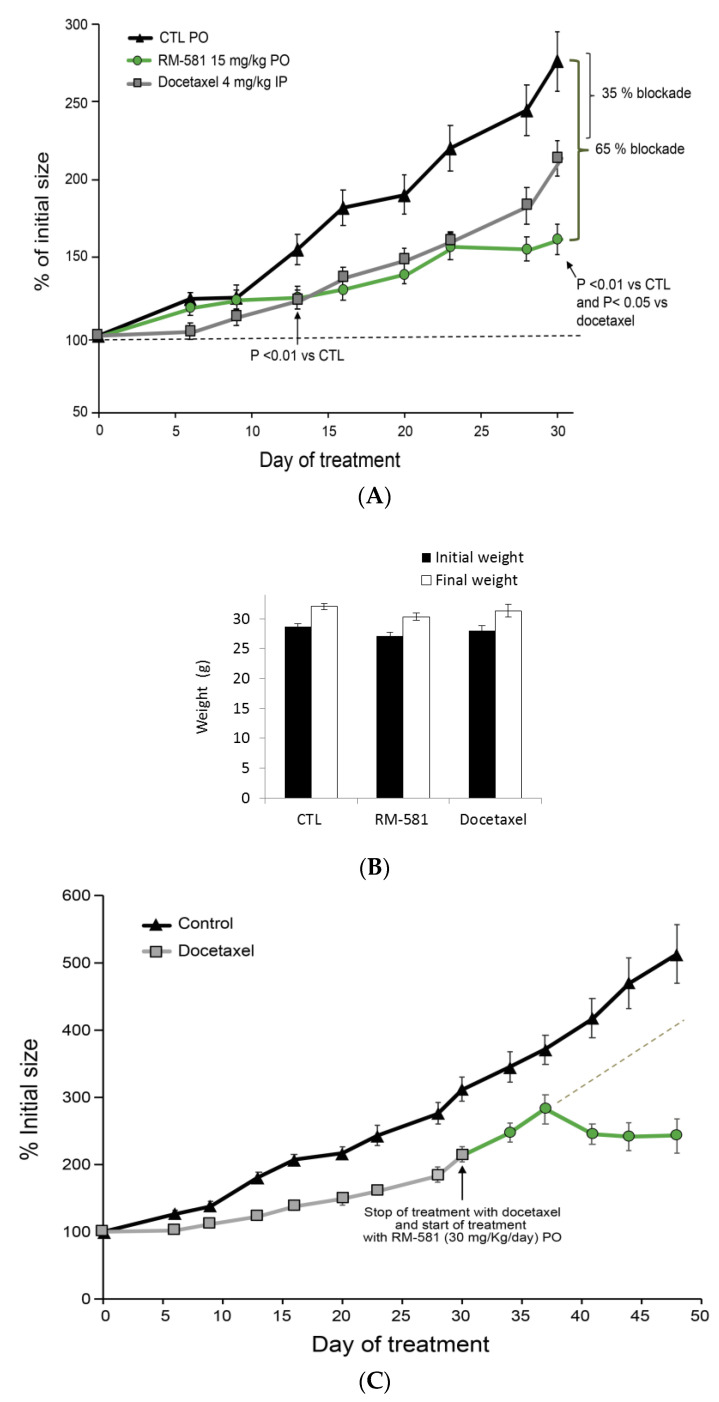

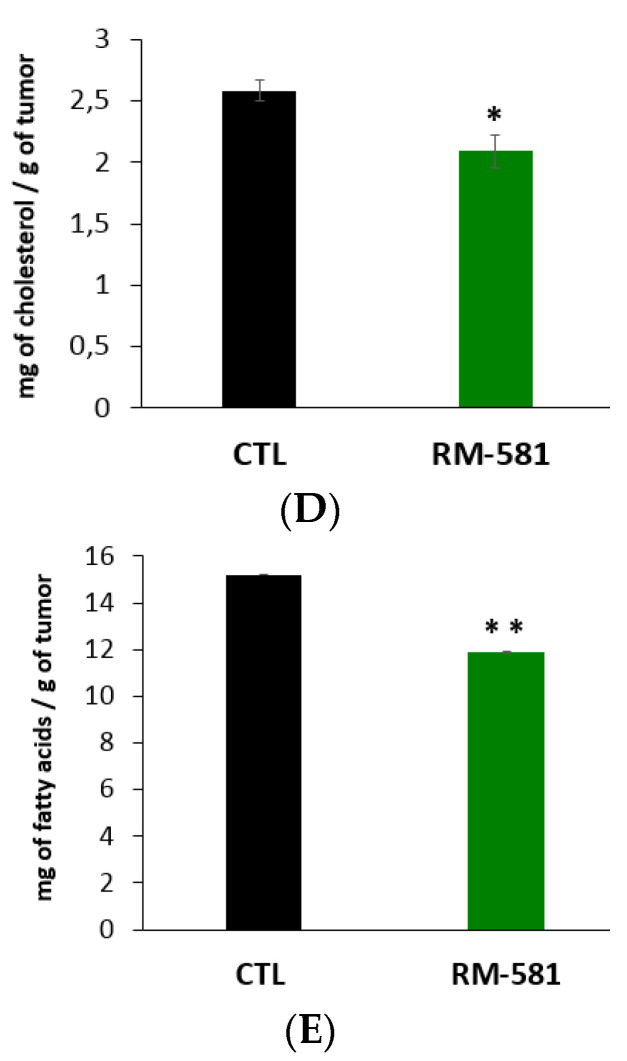
RM-581 blocks tumor growth in mice (PC-3 xenograft model). (**A**) Effects of RM-581 (15 mg/kg) administered orally (PO) 6 days per week with docetaxel (4 mg/kg) administered IP twice a week. Tumor sizes expressed as the percentage of initial tumor size (day 1 of treatment: 100%). Data are the mean ± SEM (*n* = 10–12 tumors and 6–7 mice per group). (**B**) Body weight of mice before and after treatment (30 days). Mice treated as in Figure 6A. (**C**) Effect of RM-581 on PC-3 xenograft tumors treated 30 days with docetaxel as in Figure 6A and next with RM-581, administered alone in day 38–48 at 30 mg/kg/PO, 6 days per week. Dashed line (gray) represents an extrapolation of docetaxel response from day 38 to 48. (**D**) Effect of RM-581 treatment at 15 mg/kg/day/PO during 30 days on cholesterol levels in PC-3 tumors. (**E**) Effect of RM-581 treatment at 15 mg/kg/day during 30 days on fatty acid levels in PC-3 tumors. * *p* < 0.05, ** *p* < 0.01 vs. control (CTL).

Human tumorigenic PC-3 cells were inoculated in nude mice to generate xenografts. In this in vivo cancer model, the mean tumor size of the control group nearly tripled (275%) from its initial size (100%) after 30 days (Figure 6A). However, tumor growth in mice treated orally by gavage (PO) with RM-581 (15 mg/kg) was blocked by 65% (tumor size = 155%), and RM-581 was significantly superior to docetaxel (4 mg/kg) given intraperitoneally (IP), which showed a blockade of only 35% (tumor size = 210%). The blockade effect became significant from day 13 for both treated groups. However, tumor growth blockades with docetaxel and RM-581 remained similar up to day 23, from where the response to docetaxel relapsed to an approximately half-effective blockade at day 30 compared to RM-581. Considering the toxicity of docetaxel in mice with a maximal tolerable dose of 15 mg/kg [38], and to ensure the viability of mice throughout the protocol, we used a lower dose of docetaxel (4 mg/kg/twice a week) than the 15 mg/kg/day used for RM-581. The body weight of mice was not significantly affected after 30 days of treatment in the two treated groups (Figure 6B), which agreed with previous results showing a nontoxic profile for RM-581 in mice and tissue [20,27].

The reduced effect of docetaxel in the last week of treatment (day 23 to 30) led us to suspect the arrival of resistance, a phenomenon that we observed in vitro after 11 days of treatment of PC-3 cells with docetaxel (Figure 6B). To evaluate the capacity of these docetaxel-resistant tumors to respond to RM-581, as seen in the cell proliferation assay (Figure 5C), treatment with docetaxel was stopped at day 30 and replaced by a treatment with RM-581 at 30 mg/kg/day (Figure 6C). Tumor growth was thus completely stopped at day 38 (280% of initial size) and decreased until the end of the protocol at day 48 (242% of initial size), which contrasted with the projected progression of tumor growth estimated to be around 400% for the treatment with docetaxel alone. The concentration of RM-581 in PC-3 tumors at the end of the protocol (3 h postadministration) was 3-fold higher than that in plasma (Table 1), suggesting an accumulation effect sustaining the RM-581 antitumor effect.

As complementary data to this in vivo protocol, we also measured the concentration of key lipids in PC-3 tumors after 30 days of treatment, knowing that ER stress induction could impact lipid levels [39]. As a result, RM-581 led to a significant decrease in cholesterol levels (−19.0%) and fatty acids (FA) (−21.8%) in tumors (Figure 6D,E). The level of FA represents a large panel of 56 saturated or unsaturated FA measured by GC-MS (see Table S3, Supplementary Materials, for a detailed list of all FA and their levels).

## 3. Discussion

PCa patients showing resistance to chemotherapy have very limited treatment options; thus, novel therapeutic approaches are highly needed. RM-581 showed antiproliferative activity (EC_50_ values) in the low micromolar range for different PCa cell lines, including androgen-dependent (LNCaP) and androgen-independent (DU-145 and PC-3). To evaluate the therapeutic potential of our lead AM candidate RM-581 on PCa, we selected PC-3 as a representative cell line that is nonsensitive to androgens and recognized to be one of the most aggressive PCa cell lines (metastatic) [31,32,33,40]. A first observation from the in vitro dose response proliferative assays with RM-581 in PC-3 cells (EC_50_ curve) was its capacity to block cell growth near zero at 50 µM. Flow cytometry experiments confirmed a proapoptotic effect of RM-581 at 30 µM with more than 70% of cells entering apoptosis at 72 h. The maintained activity of RM-581 in docetaxel-resistant PC-3 cells (EC_50_ = 0.9 µM) is another interesting aspect revealed by our cellular assays, showing potential for the treatment of patients who are resistant to gold-standard chemotherapy. Contrary to docetaxel, RM-581 did not induce resistance of PC-3 cells. Moreover, the combination of RM-581 with docetaxel showed a strongly synergistic effect (CI values lower than 1), offering a promising new avenue to potentiate docetaxel in the treatment of CRPC, thus reducing associated side effects by using lower doses.

In vivo evaluation in a PC-3 xenograft model in nude mice also supports the potential of RM-581 for CRPC treatment by blocking tumor progression. Our xenograft experiment showed an interesting comparative response profile to treatment with RM-581 and docetaxel. Tumor growth was slowed down by both compounds, following a very similar pattern during the first 3 weeks, but docetaxel seemed to relapse in the fourth week of treatment, progressing from 161% of initial tumor size (day 23) to 214% (day 30), in contrast with the RM-581 group, where progression was stopped during this period. The rapid tumor growth of docetaxel’s group in this last week of treatment, very close to the untreated group’s progression, led us to suspect the onset of a resistance phenomenon. We thus stopped treatment with docetaxel (day 30) and started treating mice from this group (day 31) with RM-581 (30 mg/kg/day) for 17 additional days. Following a slight increase during the first week of treatment (from 214% (day 30) to 282% (day 37)), tumor size stabilized to 214% of initial size in the second week of treatment. This offset effect, previously observed for the first week of treatment (day 1–9) in the group of mice treated with RM-581, could be caused by the time needed for the ER stress apoptosis effect to take place in tumors. Globally, the progression of tumor size when treated with RM-581 was very slight from day 31 to day 48 (from 214 to 243%) compared to the projected progression of the docetaxel group for the same period (>400%). Concomitantly, no effect on the body weight of mice was observed, suggesting a good therapeutic index.

Behind these interesting in vitro and in vivo results, the ER stress-inducing capability of RM-581 was identified as a key player. Treatment of PC-3 with 5 µM of RM-581 showed a gradual increase in the expression of 2 ER stress-related genes *BIP* (*GPR78*) and *HERP* up to 4-fold after 18 h. This result confirmed that an unfolded protein response (UPR) rapidly took place as a characteristic counteraction to ER stress [41]. In parallel, a strong elevation of *CHOP* expression, up to 8-fold, was also observed, which is an important gene marker of ER stress-related apoptosis [42]. This result is similar to previous ones observed in breast- and pancreatic-cancer cell lines [27]. In agreement with a previous study in MCF-7 cells, some RM-581-Fluo was found in ER, as highlighted from the ER colocalization experiment with an ER tracker. This confocal experiment showed the presence of RM-581 at proximity or within ER, highlighting the possibility of a direct interaction of the compound with the key proteins of ER, which could be responsible for the observed apoptotic ER stress response.

Knowing that ER stress and UPR activation affect lipid homeostasis [43], we also examined the impact of RM-581 on the biosynthesis of cholesterol and fatty acids. This was particularly important, considering that aberrant lipid metabolism was observed in PCa, and it plays an important role in cancer growth and progression [44]. Thus, two representative genes of the mevalonate (*HMGCR*) and fatty acid (*FASN*) pathways were selected as markers of these biosynthesis pathways. Treatment of PC-3 cells with RM-581 significantly downregulated their expressions, and these in vitro results were corroborated in vivo with a significant decrease in cholesterol (19%) and fatty acid (22%) levels in PC-3 tumors after 30 days of treatment. One hypothesis to explain this lipid decrease is that a massive arrival of RM-581 in ER could interact with important proteins controlling lipid synthesis [45]. Among them, cholesterol sensor INSIG–SCAP–SREBP complex is particularly interesting, considering its crucial role in the control of lipid homeostasis [46]. The stabilization of this complex by cholesterol avoids dissociation of SCAP-SREBP from INSIG and moving further to the Golgi apparatus, toward the initiation of cholesterol and fatty acid biosynthesis pathways [47]. The exogenous administration of 25-hydroxy-cholesterol decreased cholesterol biosynthesis in PC-3 cells [48]. Since RM-581 is a steroidal derivative, its sterol-like shape may favor direct or indirect interactions with proximal proteins to an SCAP–SREBP–INSIG complex, much like 25-hydroxy-cholesterol does [49], leading to the stabilization of the complex and a similar lowering response on lipid biosynthesis. This drop in lipid levels thus may somehow contribute to RM-581′s anticancer activity, but additional investigations are necessary to study this hypothesis. Therapeutic strategies against cancer based on lowering cellular cholesterol content are in clinical evaluation [50], and SCAP/SREBPs were suggested as promising metabolic targets for cancer therapy [46].

## 4. Materials and Methods

### 4.1. Chemical Synthesis

Aminosteroid RM-581 and RM-581-Fluo were prepared as previously reported, and their purity was determined by high-performance liquid chromatography as 99.6% and 91.6%, respectively [20,34].

### 4.2. Cell Culture

Human prostate cancer LNCaP, DU-145, and PC-3 cells from the American Type Culture Collection (Manassas, VA, USA) were maintained in Roswell Park Memorial Institute (RPMI)-1640 (Sigma, Saint Louis, MO, USA) containing L-glutamine (2 nmol/L) and antibiotics (100 IU penicillin/mL, 100 mg streptomycin/mL) and supplemented with 10% (*v/v*) fetal bovine serum. Cells were maintained under a 5% CO_2_ humidified atmosphere at 37 °C, and the culture medium was changed every 2–3 days. Cells were split once a week to maintain cell propagation.

### 4.3. IC_50_ Determination of RM-581 vs. RM-581-Fluo in PC-3 Cells

PC-3 cells (1 x 10^4^) were seeded in triplicate in 96-well plates in culture medium (90 µL). Cells were incubated at 37 °C in a 5% CO_2_ humidified atmosphere for 24 h. RM-581 and RM-581-Fluo were dissolved in dimethylsulfoxide (DMSO) to prepare the 1 × 10^−2^ M stock solutions. Stock solutions were diluted at multiple concentrations with culture media to obtain the final desired concentration by adding 10 µL in each well, and the mixture was incubated for 3 days. Following treatment, 20 µL of 3-(4,5-dimethylthiazol-2-yl)-5-(3-carboxymethoxyphenyl)-2-(4-sulfophenyl)-2*H*-tetrazolium (MTS) were added to each well and the mixture was incubated for 4 h. Plates were subsequently analyzed at 490 nm using a microplate reader (Molecular Devices, Sunnyvale, CA, USA), and EC_50_ values were calculated using GraphPad Prism 6 software.

### 4.4. Confocal Imaging of PC-3 Cells

The cellular localization of RM-581-Fluo was determined by evaluating its colocalization with an ER-specific dye for live-cell imaging (ER-Tracker Red (BODIPY, TR Glibenclamide), Invitrogen, Carlsbad, CA, USA) and a cell membrane stain (Cell-Mask Deep Red, Invitrogen, Carlsbad, CA, USA). For the tests, PC-3 cells were plated into 200 µL sterile Nune Lab-Tek II Chambered coverglass-8 wells using the same medium as that described above but without phenol red. After 24 h of preincubation at 37 °C to allow for the cells to properly attach, they were incubated for 2 h with compound RM-581-Fluo at a final concentration of 15 µM. ER-Tracker (1 µM final) and Cell-Mask (4.5 µg/mL) were added in the medium for the last 45 and 10 min, respectively. At the end of staining procedure, cells were placed in a Chamlide live cell system (LCI, Seoul, Republic of Korea) on an Olympus IX81 inverted microscope. The used objective was a 60X oil PlanApo N (NA 1.42). Images were acquired with the Quorum WaveFX spinning disk confocal system (Quorum Technologies, Guelph, ON, Canada) with MetaMorph version 7.8.12.0 (Molecular Devices, San Jose, CA, USA). The localization of RM-581-Fluo was performed using excitation of 405 nm, and emission filters for the green and the blue 525/50 and 460/55 nm. The same area was excited with 561 nm laser and 593/40 nm emission filter for ER specific localization; for CellMask, the wavelengths for excitation and emission were 642 and 700/75 nm, respectively. Z-stacks were acquired with at an interval of 0.2 µm between focal planes. Image analysis and deconvolution were performed with Volocity 4.0 software (Quorum Technologies, Guelph, ON, Canada).

### 4.5. Gene Expression Study (Real-Time PCR Analysis)

PC-3 cells (3 × 10^5^) were seeded in 6-well plate and treated with 2 μM of RM-581 in a time-course manner (6, 12 and 18 h). Total RNA was extracted with EZ-10 Spin Column Total RNA Mini-preps Super Kit according to the manufacturer’s instructions (Bio Basic, Markham, ON, Canada). RNA concentration was measured using a NanoDrop ND-1000 Spectrophotometer (NanoDrop Technologies, Wilmington, DE, USA). The reverse transcription reaction was performed using 5X All-In-One RT Mastermix (AbmGood, Richmond, BC, Canada) with 0.5 μg of total RNA at 42 °C for 50 min. cDNA (2.5 ng) was assessed by fluorescence-based real-time PCR quantification (qPCR), using the CFX384 Touch and SsoAdvanced Universal SYBR Green Supermix (Biorad, Mississauga, ON, Canada). A melting curve was performed to assess non-specific signals. Relative gene expressions were calculated by applying the delta Ct method, using PUM1 as a normalizing gene [51]. Primer sequences are reported in Table S4 (Supplementary Materials).

### 4.6. Apoptosis Characterization by Flow Cytometry

PC-3 cells (3 × 10^5^) were seeded overnight in triplicate in 6-well plates in culture medium (3 mL) and exposed to varying concentrations (0, 2, 10, 30, and 50 µM) of RM-581 for 6 days. After treatment, cells were collected by trypsinization and centrifugation. Collected cells were washed with ice-cold phosphate-buffered saline (PBS), and incubated with 200 µL of annexin binding buffer containing 5 µL of annexin-V fluor 488 and 4 µL of propidium iodide (0.1 mg/mL) (PI) for 12 min in the dark at room temperature. After incubation, annexin binding buffer (500 µL) was added, and cells were immediately analyzed by BD FACSCanto II (BD Bioscience, San Jose, CA, USA) by counting 28,000 events per sample. Cells were excited with a 488 nm blue laser and 633 nm red laser. Annexin-V and PI fluorescence were detected with filter set respectively at 525/50 and 585/42. The dual parametric dot plots were used to calculate the percentage of nonapoptotic live cells in the lower left quadrant (annexin-V-negative/PI-negative), early apoptotic cells in the lower right quadrant (annexin-V-positive/PI-negative), late apoptotic cells in the upper right quadrant (annexin-V-positive/PI-negative) and necrotic cells in the upper left quadrant (annexin-V-negative/PI-positive).

### 4.7. Cell Viability Proliferation Assays and Combination Index with Docetaxel

PC-3 cells (1 × 10^4^) were seeded in triplicate in 96-well plates in culture medium (90 μL). The cells were incubated at 37 °C in a 5% CO_2_ humidified atmosphere for 24 h. Docetaxel was purchased from Cayman Chemical (Ann Arbor, MI, USA), gemcitabine, oxaliplatin, 5-fluorouracil and irinotecan were purchased from Ark Pharm (Arlington Heights, IL, USA) and cisplatin was purchased from EMD Millipore (Etobicoke, ON, Canada). The antineoplastic compounds were dissolved in DMSO (10 mm) and stock solutions were diluted at multiple concentrations with culture media to obtain the desired final concentration by adding 10 μL in each well, and the mixture was incubated for 3 days. The cell proliferation assay was performed using (MTS) (Cell Titer 96 Aqueous, Promega, Nepean, ON, Canada). Following treatment, MTS (20 μL) was added to each well and the mixture was incubated for 4 h. The plates were subsequently analyzed at 490 nm using a Tecan M-200 microplate reader (Männedorf, Switzerland), and EC_50_ values were calculated using GraphPad Prism 7 software. Values represent the average of two independent experiments preformed in triplicate. The combination index (CI) was calculated using the Chou–Talalay method [37] according to the following formula:

$$\mathrm{CI}=\frac{{\left(D\right)}_{1}}{{\left(D_{X\;}\right)}_{1}}+\frac{{\left(D\right)}_{2}}{{\left(D_{X\;}\right)}_{2}}$$

where denominators (Dx)_l_ and (Dx)_2_ are the doses of the individual drug required to achieve a given effect level, and numerators (D)_1_ and (D)_2_ are the concentrations of each drug present in combination to trigger the same effect level. When the drugs additively interact, CI = 1, CI < 1 indicates a synergistic interaction and CI > 1 indicates an antagonistic effect.

### 4.8. Induction of Docetaxel-Resistant PC-3 Cells

Docetaxel-resistant PC-3 sub-cell line (PC-3_dx_) was generated by initially treating cells with docetaxel (10 µM) in 75 cm^2^ flasks for 11 days and the culture medium was changed every 2–3 days. Resistance was determined on the basis of decreased cell death and increased cell proliferation. After this treatment, the surviving cells (1 × 10^4^) were seeded in triplicate in 96-well plates in culture medium (90 µL) and treated as mentioned above.

### 4.9. Effect of RM-581 in PC-3 Xenografts

Xenograft experiments were performed following a protocol that had been approved by our Institutional Animal Care Committee (Comité de protection des animaux de l’Université Laval, Québec, QC, Canada), and conducted in an animal facility approved by the Canadian Council on Animal Care (CCAC). Male Balb/c athymic nude mice weighing approximately 28 g were obtained from Charles River Inc. (Saint-Constant, QC, Canada) and housed (4 to 5) in vinyl microisolated ventilated cages equipped with air lids, which were kept in laminar airflow hoods and maintained under pathogen-limiting conditions. During the acclimatization and study period, the animals were housed under a controlled environment at 22 ± 3 °C, with 50 ± 20% relative humidity and light set at 12 h/day (light on at 07:15). Sterile rodent food (Rodent diet #T.2018.15, Harlan Teklad, Madison, WI, USA) and water were provided ad libitum. After 5 days of acclimatization, the mice were inoculated by subcutaneous (SC) injection of 1 × 10^6^ PC-3 cells (Passage 58) in 0.1 mL of growth medium containing 30% Matrigel (BD Biosciences, Mississauga, ON, Canada) into both flanks via a 2.5-cm long 22-gauge needle. After 2 weeks, mice with tumors were randomized according to tumor volume, separated into three groups, weighed, and treated over a period of 30 days or 48 days.

For the control group (Gr 1), 8 mice (15 tumors) were treated with 0.1 mL of vehicle alone (8% DMSO and 92% propylene glycol (PG)) administrated orally (PO) by gavage 6 days a week until day 48. For the RM-581-treated group (Gr 2), 8 mice (16 tumors) received RM-581 (0.416 mg suspended in 0.1 mL of vehicle; 15 mg/kg on average) PO 6 days a week until day 30. For the docetaxel-treated group (Gr 3), 8 mice (15 tumors) received docetaxel (0.111 mg suspended in 0.1 mL of vehicle; 4 mg/kg on average) intraperitoneally twice a week until day 30. Thereafter, docetaxel was replaced by RM-581 (30 mg/kg/PO) until day 48. Both compounds were first dissolved in DMSO, and thereafter we added PG to obtain the appropriate concentration in the vehicle fluid (0.1 mL injected). All solutions were prepared one day prior to the initiation of treatment, stored at 4 °C, and kept under constant agitation.

Tumor size was measured twice a week with a caliper. Two perpendicular diameters (*L* and *W*) were recorded, and tumor area (mm^2^) was calculated using formula *L*/2 × *W*/2 × π. The measured area on the first day of treatment was taken as 100%. At the end of the studies and 3 h after the last treatment, mice were terminally anaesthetized, and final body weights were measured. During necropsy, tumors, liver, kidney, and total prostate, and blood by cardiac puncture were collected from mice and immediately frozen at −80 °C until analysis.

### 4.10. Dosage of RM-581 in Tumors and Plasma of Treated Mice

The concentration of RM-581 was determined by liquid chromatography–mass spectrometry/mass spectrometry (LC−MS/MS) analysis using a procedure developed at the CHU de Québec-Research Center (Québec, Canada) (Bioanalytical Service) and previously published [52].

### 4.11. Fatty Acid Determination in PC-3 Tumors

A solution of chloroform and methanol (2:1, *v/v*) was used to extract lipids from 80 mg of tissue, as previously described [53]. The fatty acid profile was next determined by gas chromatography (GC), coupled with an ionization detector, and using phosphatidylcholine C21:0 (Avanti Polar Lipids) as an internal standard. GC model 5890 was from Agilent Technologies (Mississauga, ON, Canada). The used capillary column was a HP-88 (100 m × 0.25 mm, 0.2 mm, Agilent Technologies), and the carrier gas was helium at 1.5 mL/min. Fatty acids were identified according to their retention time using fatty acid mixtures as standards [54].

### 4.12. Statistical Analysis

Statistical significance was determined according to the Duncan–Kramer multiple-range test [55]. Other differences were evaluated using a *t*-test; *p* values of less than 0.05 were considered to be statistically significant.

## 5. Conclusions

RM-581 targets the ER, trigger ER stress-related apoptosis, and decreases lipid biosynthesis in PC-3 cells and tumors. By inhibiting cell proliferation in PC-3 cells and docetaxel-resistant PC-3 cells, RM-581 represents a promising selective anticancer agent showing a good toxicity profile and strong potential for combination with other antineoplastic drugs. This work also highlights the potential of an ER stress inducer for the treatment of prostate-cancer types that are refractory to current chemotherapy.

## Figures and Tables

**Table 1:** Tumor and plasma concentrations of RM-581 from xenograft experiments as determined by LC-MS/MS at the end of the protocol (3 h post-treatment).

RM-581 Tumor (ng/g)	RM-581 Plasma (ng/mL)	Concentration Index * Tumor/Plasma
150 ± 71	50.5 ± 6.3	3.0

(*) For the tumor concentration of RM-581, we estimated ng/g = ng/mL.

## Data Availability

Data are contained within the article or supplementary materials.

## Supplementary Materials

All data are available online at https://www.mdpi.com/article/10.3390/ijms222011181/s1. Tables S1–S4 and Figures S1–S4.

## References

[1] Siegel Rebecca L., Miller Kimberly D., Jemal A. (2017). Cancer statistics, 2017. CA Cancer J. Clin..

[2] Kim M.M., Hoffman K.E., Levy L.B., Frank S.J., Pugh T.J., Choi S., Nguyen Q.N., McGuire S.E., Lee A.K., Kuban D.A. (2012). Improvement in prostate cancer survival over time: A 20-year analysis. Cancer J..

[3] Karantanos T., Corn P.G., Thompson T.C. (2013). Prostate cancer progression after androgen deprivation therapy: Mechanisms of castrate resistance and novel therapeutic approaches. Oncogene.

[4] Scher H.I., Solo K., Valant J., Todd M.B., Mehra M. (2015). Prevalence of prostate cancer clinical states and mortality in the United States: Estimates using a dynamic progression model. PLoS ONE.

[5] Cornford P., Bellmunt J., Bolla M., Briers E., De Santis M., Gross T., Henry A.M., Joniau S., Lam T.B., Mason M.D. (2017). EAU-ESTRO-SIOG Guidelines on Prostate Cancer. Part II: Treatment of relapsing, metastatic, and castration-resistant prostate cancer. Eur. Urol..

[6] Holm H.V., Dahl A.A., Klepp O.H., Fossa S.D. (2017). Modern treatment of metastatic prostate cancer. Tidsskr. Nor. Laegeforen.

[7] Helsen C., van den Broeck T., Voet A., Prekovic S., van Poppel H., Joniau S., Claessens F. (2014). Androgen receptor antagonists for prostate cancer therapy. Endocr. Relat. Cancer.

[8] Ritch C.R., Cookson M.S. (2016). Advances in the management of castration resistant prostate cancer. BMJ.

[9] Smith M.R., Kabbinavar F., Saad F., Hussain A., Gittelman M.C., Bilhartz D.L., Wynne C., Murray R., Zinner N.R., Schulman C. (2005). Natural history of rising serum prostate-specific antigen in men with castrate nonmetastatic prostate cancer. J. Clin. Oncol..

[10] Berthold D.R., Pond G.R., Soban F., de Wit R., Eisenberger M., Tannock I.F. (2008). Docetaxel plus prednisone or mitoxantrone plus prednisone for advanced prostate cancer: Updated survival in the TAX 327 study. J. Clin. Oncol..

[11] Petrylak D.P., Tangen C.M., Hussain M.H., Lara P.N., Jones J.A., Taplin M.E., Burch P.A., Berry D., Moinpour C., Kohli M. (2004). Docetaxel and estramustine compared with mitoxantrone and prednisone for advanced refractory prostate cancer. N. Engl. J. Med..

[12] Fizazi K., Scher H.I., Molina A., Logothetis C.J., Chi K.N., Jones R.J., Staffurth J.N., North S., Vogelzang N.J., Saad F. (2012). Abiraterone acetate for treatment of metastatic castration-resistant prostate cancer: Final overall survival analysis of the COU-AA-301 randomised, double-blind, placebo-controlled phase 3 study. Lancet Oncol..

[13] Merseburger A.S., Haas G.P., von Klot C.A. (2015). An update on enzalutamide in the treatment of prostate cancer. Ther. Adv. Urol..

[14] Fernandez O., Afonso J., Vazquez S., Campos B., Lazaro M., Leon L., Anton Aparicio L.M. (2014). Metastatic castration-resistant prostate cancer: Changing landscape with cabazitaxel. Anticancer Drugs.

[15] Fryzek J.P., Reichert H., Summers N., Townes L., Deuson R., Alexander D.D., Vanderpuye-Orgle J. (2018). Indirect treatment comparison of cabazitaxel for patients with metastatic castrate-resistant prostate cancer who have been previously treated with a docetaxel-containing regimen. PLoS ONE.

[16] Brasso K., Thomsen F.B., Schrader A.J., Schmid S.C., Lorente D., Retz M., Merseburger A.S., von Klot C.A., Boegemann M., de Bono J. (2015). Enzalutamide antitumour activity against metastatic castration-resistant prostate cancer previously treated with docetaxel and abiraterone: A multicentre analysis. Eur. Urol..

[17] Culig Z. (2017). Molecular Mechanisms of Enzalutamide Resistance in Prostate Cancer. Curr. Mol. Biol. Rep..

[18] Thelen P., Gschwend J., Wolff J.M., Miller K. (2016). Mechanisms of resistance in antihormone therapies of advanced prostate cancer. Aktuelle Urol.

[19] Yoo S., Choi S.Y., You D., Kim C.-S. (2016). New drugs in prostate cancer. Prostate Int..

[20] Perreault M., Maltais R., Roy J., Dutour R., Poirier D. (2017). Design of a mestranol 2-N-piperazino-Ssubstituted serivative showing potent and selective in vitro and in vivo activities in MCF-7 breast cancer models. ChemMedChem.

[21] Maltais R., Hospital A., Delhomme A., Roy J., Poirier D. (2014). Chemical synthesis, NMR analysis and evaluation on a cancer xenograft model (HL-60) of the aminosteroid derivative RM-133. Steroids.

[22] Perreault M., Maltais R., Dutour R., Poirier D. (2016). Explorative study on the anticancer activity, selectivity and metabolic stability of related analogs of aminosteroid RM-133. Steroids.

[23] Kenmogne L.C., Ayan D., Roy J., Maltais R., Poirier D. (2015). The aminosteroid derivative RM-133 shows in vitro and in vivo antitumor activity in human ovarian and pancreatic cancers. PLoS ONE.

[24] Jegham H., Maltais R., Dufour P., Roy J., Poirier D. (2012). Solid-phase chemical synthesis and in vitro biological evaluation of novel 2β-piperazino-(20R)-5α-pregnane-3α,20-diol N-derivatives as anti-leukemic agents. Steroids.

[25] Jegham H., Maltais R., Roy J., Doillon C., Poirier D. (2012). Biological evaluation of a new family of aminosteroids that display a selective toxicity for various malignant cell lines. Anticancer Drugs.

[26] Perreault M., Maltais R., Kenmogne L.K., Létourneau D., Gobeil S., LeHoux J.-G., Poirier D. (2018). Implication of STARD5 and cholesterol homeostasis disturbance in the endoplasmic reticulum stress-related response induced by pro-apoptotic aminosteroid RM-133. Pharmacol. Res..

[27] Perreault M., Maltais R., Roy J., Picard S., Popa I., Bertrand N., Poirier D. (2019). Induction of endoplasmic reticulum stress by aminosteroid derivative RM-581 leads to tumor regression in PANC-1 xenograft model. Investig. New Drugs.

[28] Dutour R., Maltais R., Perreault M., Roy J., Poirier D. (2018). Parallel solid-phase synthesis using a new diethylsilylacetylenic linker and leading to mestranol derivatives with potent antiproliferative activities on multiple cancer cell lines. Anticancer Agents Med. Chem..

[29] Yadav R.K., Chae S.W., Kim H.R., Chae H.J. (2014). Endoplasmic reticulum stress and cancer. J. Cancer Prev..

[30] Storm M., Sheng X., Arnoldussen Y.J., Saatcioglu F. (2016). Prostate cancer and the unfolded protein response. Oncotarget.

[31] Cunningham D., You Z. (2015). In vitro and in vivo model systems used in prostate cancer research. J. Biol. Methods.

[32] Atala A. (2012). Re: PC3 is a cell line characteristic of prostatic small cell carcinoma. J. Urol..

[33] Kaighn M.E., Narayan K.S., Ohnuki Y., Lechner J.F., Jones L.W. (1979). Establishment and characterization of a human prostatic carcinoma cell line (PC-3). Invest. Urol..

[34] Maltais R., Roy J., Poirier D. (2021). Turning a quinoline-based steroidal anticancer agent into fluorescent dye for its tracking by cell imaging. ACS Med. Chem. Lett..

[35] Fribley A., Zhang K., Kaufman R.J. (2009). Regulation of apoptosis by the unfolded protein response. Methods Mol. Biol..

[36] Nagelkerke A., Bussink J., Sweep F.C.G.J., Span P.N. (2014). The unfolded protein response as a target for cancer therapy. Biochim. Biophys. Acta BBA Rev. Cancer.

[37] Wang M., Kaufman R.J. (2014). The impact of the endoplasmic reticulum protein-folding environment on cancer development. Nat. Rev. Cancer.

[38] Chou T.-C. (2010). Drug Combination studies and their synergy quantification using the Chou-Talalay method. Cancer Res..

[39] Dykes D.J., Bissery M.C., Harrison S.D., Waud W.R. (1995). Response of human tumor xenografts in athymic nude mice to docetaxel (RP 56976, Taxotere®). Invest. New Drugs.

[40] Pineau L., Colas J., Dupont S., Beney L., Fleurat-Lessard P., Berjeaud J.M., Bergès T., Ferreira T. (2009). Lipid-induced ER stress: Synergistic effects of sterols and saturated fatty acids. Traffic.

[41] Yu Y., Yang F.H., Zhang W.T., Guo Y.D., Ye L., Yao X.D. (2021). Mesenchymal stem cells desensitize castration-resistant prostate cancer to docetaxel chemotherapy via inducing TGF-β1-mediated cell autophagy. Cell Biosci..

[42] Hetz C. (2012). The unfolded protein response: Controlling cell fate decisions under ER stress and beyond. Nat. Rev. Mol. Cell Biol..

[43] Li Y., Guo Y., Tang J., Jiang J., Chen Z. (2014). New insights into the roles of CHOP-induced apoptosis in ER stress. Acta Biochim. Et Biophys. Sin..

[44] Deep G., Schlaepfer I.R. (2016). Aberrant lipid metabolism promotes prostate cancer: Role in cell survival under hypoxia and extracellular vesicles biogenesis. Int. J. Mol. Sci..

[45] Colgan S.M., Hashimi A.A., Austin R.C. (2011). Endoplasmic reticulum stress and lipid dysregulation. Expert Rev. Mol. Med..

[46] Cheng X., Li J., Guo D. (2018). SCAP/SREBPs are central players in lipid metabolism and novel metabolic targets in cancer therapy. Curr. Top. Med. Chem..

[47] DeBose-Boyd R.A., Brown M.S., Li W.P., Nohturfft A., Goldstein J.L., Espenshade P.J. (1999). Transport-dependent proteolysis of SREBP: Relocation of site-1 protease from Golgi to ER obviates the need for SREBP transport to Golgi. Cell.

[48] Krycer J.R., Kristiana I., Brown A.J. (2010). Cholesterol homeostasis in two ommonly used human prostate cancer cell-lines, LNCaP and PC-3. PLoS ONE.

[49] Adams C.M., Reitz J., De Brabander J.K., Feramisco J.D., Li L., Brown M.S., Goldstein J.L. (2004). Cholesterol and 25-hydroxycholesterol inhibit activation of SREBPs by different mechanisms, both involving SCAP and Insigs. J. Biol. Chem..

[50] Gu L., Saha S.T., Thomas J., Kaur M. (2019). Targeting cellular cholesterol for anticancer therapy. FEBS J..

[51] Pfaffl M.W. (2001). A new mathematical model for relative quantification in real-time RT-PCR. Nucleic Acids Res..

[52] Cortés-Benítez F., Roy J., Perreault M., Maltais R., Poirier D. (2019). A- and D-ring structural modifications of an androsterone derivative inhibiting 17β-hydroxysteroid dehydrogenase type 3: Chemical synthesis and structure-activity relationships. J. Med. Chem..

[53] Moreel X., Allaire J., Leger C., Caron A., Labonte M.E., Lamarche B., Julien P., Desmeules P., Tetu B., Fradet V. (2014). Prostatic and dietary omega-3 fatty acids and prostate cancer progression during active surveillance. Cancer Prev. Res..

[54] Gevariya N., Besancon M., Robitaille K., Picard V., Diabate L., Alesawi A., Julien P., Fradet Y., Bergeron A., Fradet V. (2019). Omega-3 fatty acids decrease prostate cancer progression associated with an anti-tumor immune response in eugonadal and castrated mice. Prostate.

[55] Kramer C.Y. (1956). Extension of multiple range tests to roup means with unequal numbers of replications. Biometrics.

