# Supplementary material for: Induction of Endoplasmic Reticulum Stress-Mediated Apoptosis by Aminosteroid RM-581 Efficiently Blocks the Growth of PC-3 Cancer Cells and Tumors Resistant or Not to Docetaxel
Source: Int J Mol Sci. 2021 Oct 17;22(20):11181. doi: 10.3390/ijms222011181 (PMC8537847; doi:10.3390/ijms222011181)

Supplementary Materials

Induction of endoplasmic reticulum stress-mediated apoptosis by aminosteroid RM-581 efficiently blocks the growth of PC-3 docetaxel-resistant prostate cancer cells and tumors

René Maltais [1], Jenny Roy [1], Martin Perreault [1], Sachiko Sato [2], Julie-Christine Lévesque [2], and Donald Poirier [1,3,*]

[1] *Laboratory of Medicinal Chemistry, Endocrinology and Nephrology Unit, CHU de Québec Research Center, Québec, QC, G1V 4G2, Canada.*
[2] *Bioimaging platform, CHUL-CHU de Quebec Research Centre, Faculty of Medicine, Laval University, Québec, QC, G1V 4G2, Canada.*
[3] *Department of Molecular Medicine, Faculty of Medicine, Université Laval, Québec, QC, G1V 0A6, Canada.*

Supplementary Materials

Table of contents... 1
Table S1. Calculated physicochemical and ADME properties of RM-581-Fluo.......................... 2
Table S2. Calculated physicochemical and ADME properties of RM-581 3
Table S3. List of fatty acids dosed in PC-3 tumors... 4
Table S4. Primer sequences... 6

Figure S1. Cell proliferation and EC_{50} values in LNCaP (**A**), DU-145 (**B**) and PC-3 (**C**) cells treated with RM-581 ……………………………………………………………...… 7
Figure S2. Emission spectra of RM-581-Fluo at different pH and concentrations ……..…….… 8
Figure S3. Three additional confocal imaging pictures from a second experiment with RM-581-Fluo in PC-3 cells ………………………………………………………….... 10
Figure S4. Cell proliferation and EC_{50} values after continuous exposure to RM-581 in PC-3 cells ………………………………………………………………………….........13

Table S1. Calculated physicochemical and ADME properties of RM-581-Fluo

[http://www.swissadme.ch/faq.php]. Copyright [2021] [SwissADME].

Molecule 1

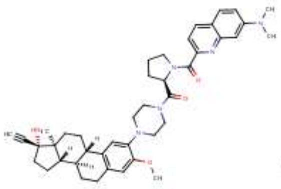

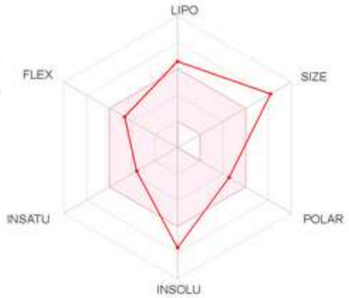

SMILES	C#C[C@]1(O)CC[C@@H]2[C@]1(C)CC[C@H]1[C@H]2CCc2c1cc(N1CCN(CC1)C(=O)[C@H]1CCCN1C(=O)c1ccc3c(n1)cc(cc3)N(C)C)c(c2)OC
**Physicochemical Properties**	
Formula	C42H51N5O4
Molecular weight	689.89 g/mol
Num. heavy atoms	51
Num. arom. heavy atoms	16
Fraction Csp3	0.55
Num. rotatable bonds	7
Num. H-bond acceptors	5
Num. H-bond donors	1
Molar Refractivity	212.92
TPSA	89.45 Å²
**Lipophilicity**	
Log $P_{o/w}$ (iLOGP)	5.31
Log $P_{o/w}$ (XLOGP3)	5.96
Log $P_{o/w}$ (WLOGP)	4.42
Log $P_{o/w}$ (MLOGP)	3.67
Log $P_{o/w}$ (SILICOS-IT)	5.02
Consensus Log $P_{o/w}$	4.88

**Water Solubility**	
Log *S* (ESOL)	-7.64
Solubility	1.57e-05 mg/ml ; 2.28e-08 mol/l
Class	Poorly soluble
Log *S* (Ali)	-7.61
Solubility	1.68e-05 mg/ml ; 2.43e-08 mol/l
Class	Poorly soluble
Log *S* (SILICOS-IT)	-8.15
Solubility	4.88e-06 mg/ml ; 7.08e-09 mol/l
Class	Poorly soluble
**Pharmacokinetics**	
GI absorption	High
BBB permeant	No
P-gp substrate	No
CYP1A2 inhibitor	No
CYP2C19 inhibitor	Yes
CYP2C9 inhibitor	No
CYP2D6 inhibitor	No
CYP3A4 inhibitor	Yes
Log K_p (skin permeation)	-6.28 cm/s
**Druglikeness**	
Lipinski	Yes; 1 violation: MW>500
Ghose	No; 3 violations: MW>480, MR>130, #atoms>70
Veber	Yes
Egan	Yes
Muegge	No; 3 violations: MW>600, XLOGP3>5, #rings>7
Bioavailability Score	0.55
**Medicinal Chemistry**	
PAINS	0 alert
Brenk	1 alert: triple_bond
Leadlikeness	No; 2 violations: MW>350, XLOGP3>3.5
Synthetic accessibility	6.30

Table S2. Calculated physicochemical and ADME properties of RM-581

[http://www.swissadme.ch/faq.php]. Copyright [2021] [SwissADME].

Molecule 1

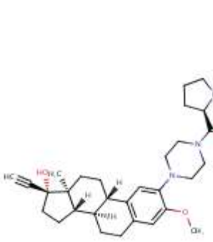

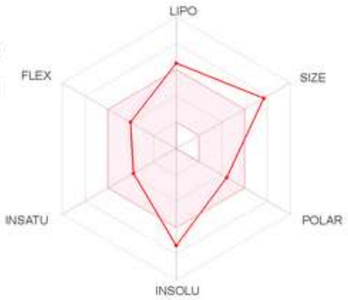

SMILES: COc1cc2CC[C@@H]3[C@@H](c2cc1N1CCN(CC1)C(=O)[C@H]1CCCN1C(=O)c1ccc2c(n1)cccc2)CC[C@]1([C@H]3CC[C@@]1(O)C#C)C

Physicochemical Properties	
Formula	C40H46N4O4
Molecular weight	646.82 g/mol
Num. heavy atoms	48
Num. arom. heavy atoms	16
Fraction Csp3	0.53
Num. rotatable bonds	6
Num. H-bond acceptors	5
Num. H-bond donors	1
Molar Refractivity	198.71
TPSA	86.21 Å²

Lipophilicity	
Log $P_{o/w}$ (iLOGP)	5.14
Log $P_{o/w}$ (XLOGP3)	5.84
Log $P_{o/w}$ (WLOGP)	4.35
Log $P_{o/w}$ (MLOGP)	3.85
Log $P_{o/w}$ (SILICOS-IT)	5.28
Consensus Log $P_{o/w}$	4.89

Water Solubility	
Log *S* (ESOL)	-7.38
Solubility	2.70e-05 mg/ml ; 4.17e-08 mol/l
Class	Poorly soluble
Log *S* (Ali)	-7.42
Solubility	2.45e-05 mg/ml ; 3.79e-08 mol/l
Class	Poorly soluble
Log *S* (SILICOS-IT)	-8.10
Solubility	5.13e-06 mg/ml ; 7.93e-09 mol/l
Class	Poorly soluble

Pharmacokinetics	
GI absorption	High
BBB permeant	No
P-gp substrate	No
CYP1A2 inhibitor	No
CYP2C19 inhibitor	Yes
CYP2C9 inhibitor	No
CYP2D6 inhibitor	No
CYP3A4 inhibitor	Yes
Log K_p (skin permeation)	-6.10 cm/s

Druglikeness	
Lipinski	Yes; 1 violation: MW>500
Ghose	No; 3 violations: MW>480, MR>130, #atoms>70
Veber	Yes
Egan	Yes
Muegge	No; 3 violations: MW>600, XLOGP3>5, #rings>7
Bioavailability Score	0.55

Medicinal Chemistry	
PAINS	0 alert
Brenk	1 alert: triple_bond
Leadlikeness	No; 2 violations: MW>350, XLOGP3>3.5
Synthetic accessibility	5.92

Table S3. List of fatty acids dosed in human PC-3 tumors

		CTL group (PC-3 tumor)		RM-581 (PC-3 tumor)	
**Fatty acids (FA)**	**Common name**	**%**	**mg FA/g of tissue**	**%**	**mg FA/g of tissue**
**C8:0**		0.00	0.00	0.00	0.00
**C10:0**		0.00	0.00	0.00	0.00
**C12:0**		0.17	0.03	0.15	0.02
**14:0**	Myristic acid	1.48	0.22	1.35	0.16
9t-**14:1 n5**		0.00	0.00	0.00	0.00
9c-**14:1 n5**	Myristoleic acid	0.15	0.02	0.12	0.01
**C15:0**		0.15	0.02	0.17	0.02
**C16:0 DMA**		0.00	0.00	0.04	0.01
**C15:1 n5t**		0.17	0.03	0.19	0.02
**C15:1 n5c**		0.00	0.00	0.00	0.00
**C15:1 n1c**		0.00	0.00	0.00	0.00
**16:0**	Palmitic acid	19.59	2.98	18.71	2.22
9t-**16:1 n7**	Palmitelaidic acid	0.00	0.00	0.00	0.00
**C17:0 isobranched**		0.00	0.00	0.00	0,00
9c-**16:1 n7**	Palmitoleic acid	9.87	1.50	6.96	0.83
**C18:0 DMA**		0.00	0.00	0.00	0.00
**C17:1 n7t**		0.12	0.02	0.13	0.02
**C18:1 DMA**		0.18	0.03	0.20	0.02
**C17:1 n7c**		0.00	0.00	0.00	0.00
**18:0**	Stearic acid	2.77	0.42	3.30	0.39
6t-**18:1 n12**	Petroselaidic acid	0.00	0.00	0.00	0.00
9t-**18:1 n9**	Elaidic acid	0.01	0.00	0.00	0.00
11t-**18:1 n7**	Transvaccenic acid	0.07	0.01	0.13	0.02
7c-**18:1 n11**/6c-**18:1 n12**	/Petroselinic acid	0.00	0.00	0.00	0.00
9c-**18:1 n9**	Oleic acid	31.44	4.78	30.38	3.61
11c-**18:1 n7**	Vaccenic acid	2.05	0.31	2.05	0.24
12c-**18:1 n6**		0.00	0.00	0.00	0.00
13c-**18:1 n5**		0.03	0.00	0.03	0.00
9t12t-**18:2 n6**	Linolelaidic acid	0.00	0.00	0.00	0.00
9c12t-**18:2 n6**		0.09	0.01	0.11	0.01
9t12c-**18:2 n6**		0.00	0.00	0.00	0.00
9c12c-**18:2 n6** (LA)	Linoleic acid	26.27	3.99	28.90	3.44
9t12t15t-**18:3 n3**		0.06	0.01	0.04	0.00
**20:0**	Arachidic acid	0.19	0.03	0.25	0.03
6c9c12c-**18:3 n6**	gamma-Linolenic acid	0.06	0.01	0.06	0.01
9c12c15c-**18:3 n3** (ALA)	alpha-Linolenic acid	1.18	0.18	1.25	0.15
8c-**20:1 n12**		0.00	0.00	0.00	0,00
11c-**20:1 n9**	Gondoic acid	0.58	0.09	0.61	0.07

6c9c12c15c-**18:4 n3**	Stearidonic acid	0.04	0.01	0.04	0.00
11c14c-**20:2 n6**		0.31	0.05	0.48	0.06
**22:0**	Behenic acid	0.08	0.01	0.09	0.01
8c11c14c-**20:3 n6**	Dihomo-gamma-linolenic acid	0.47	0.07	0.76	0.09
**C22:1 n9t**		0.00	0.00	0.00	0.00
11c14c17c-**20:3 n3** (ETE)		0.02	0.00	0.03	0.00
5c8c11c14c-**20:4 n6** (AA)	Arachidonic acid	0.71	0.11	1.04	0.12
13c-**22:1 n9**	Erucic acid	0.07	0.01	0.08	0.01
8c11c14c17c-**20:4 n3** (ETA)		0.01	0.00	0.04	0.00
13c16c-**22:2 n6**		0.03	0.00	0.05	0.01
5c8c11c14c17c-**20:5 n3** (EPA)	Timnodonic acid	0.02	0.00	0.02	0.00
**24:0**	Lignoceric acid	0.30	0.05	0.32	0.04
13c16c19c-**22:3 n3**		0.00	0.00	0.00	0.00
15c-**24:1 n9**	Nervonic acid	0.05	0.01	0.06	0.01
7c10c13c16c-**22:4 n6**	Adrenic acid	0.54	0.08	0.82	0.10
4c7c10cx13c16c-**22:5 n6**	Adrenic acid	0.14	0.02	0.20	0.02
7c10c13c16c19c-**22:5 n3** (DPA)		0.15	0.02	0.22	0.03
4c7c10c13c16c19c-**22:6 n3** (DHA)	Cervonic acid	0.37	0.06	0.58	0.07
**Total**		100.00	15.20	100.00	11.89

**n-3/n-6 (0,20 à0,33)**	0.06	0.06	0.07	0.07
**n-6 total**	28.63	4.35	32.42	3.85
**n-3 total**	1.81	0.28	2.18	0.26

**Saturated**	24.73	3.76	24.40	2.90
**P (n-3 & 6)/S**	1.23	1.23	1.42	1.42
**Polyunsaturated n3 cis**	1.75	0.27	2.14	0.25
**Polyunsaturated n6 cis**	28.52	4.33	32.29	3.84
**Polyunsaturated n3 trans**	0.06	0.01	0.04	0.00
**Polyunsaturated n6 trans**	0.09	0.01	0.11	0.01
**Monounsaturated trans**	0.37	0.06	0.44	0.05
**Monounsaturated cis**	44.24	6.72	40.29	4.79
**Monounsaturated n6 cis**	0.00	0.00	0.00	0.00
**Total trans**	0.52	0.08	0.60	0.07
**Total cis**	74.57	11.33	74.77	8.89

**Total of omega-6 cis**	28.54	4.34	32.31	3.84

Table S4. Primer sequences

	Forward sequence	Reverse sequence
HMGCR	TTCGGTGGCCTCTAGTGAGA	AAAGCTTCATTCAAGCCTGTCA
FASN	ATGGAGGAGGTGGTGATTGC	CTGGGCCCTCTGAAGTCGAA
BIP	CTTGGTATTGAAACTGTGGGAGGTG	TTCCAGTCAGATCAAATGTACCCAG
CHOP	GGAGGAGCCAGAACCAGCAGA	TTCCGTTTCCTGGTTCTCCCTT
HERP	TGCATCAGGGGCTTTTGTTCC	AACCACTTGAGGAGCAGCATTCT

Figure S1. Cell proliferation and EC_{50} values in LNCaP (**A**), DU-145 (**B**) and PC-3 (**C**) cells treated with RM-581. EC_{50} values of 1.2, 4.4 and 1.2 µM were obtained for LNCaP, DU-145 and PC-3 cells, respectively.

A) LNCaP Cells

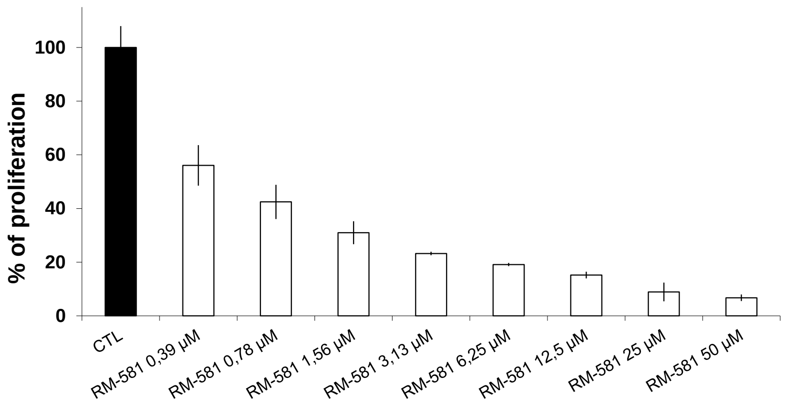

B) DU-145 Cells

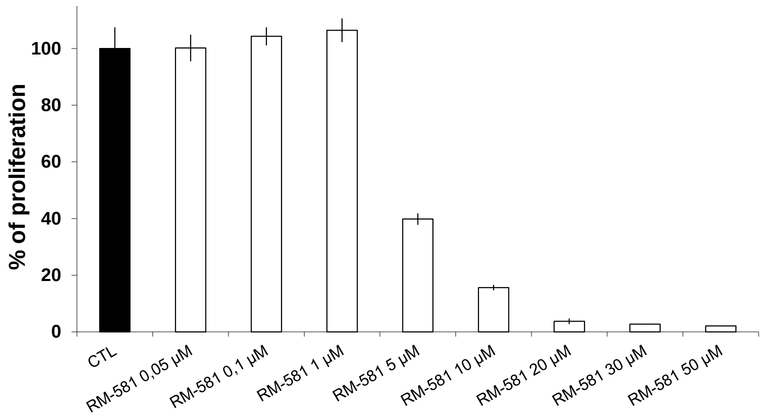

C) PC-3 Cells

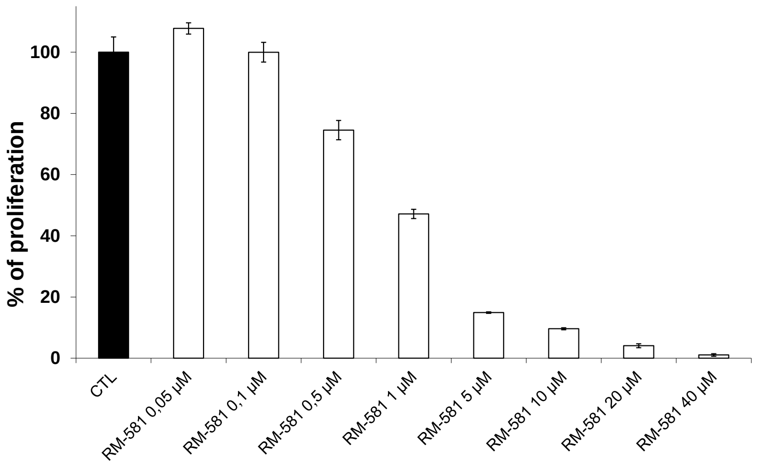

Figure S2. Emission spectra of RM-581-Fluo at different pH and concentrations

a) Phosphate buffer, pH 7.5, RM-581-Fluo (50 µM), Excitation 405 nm

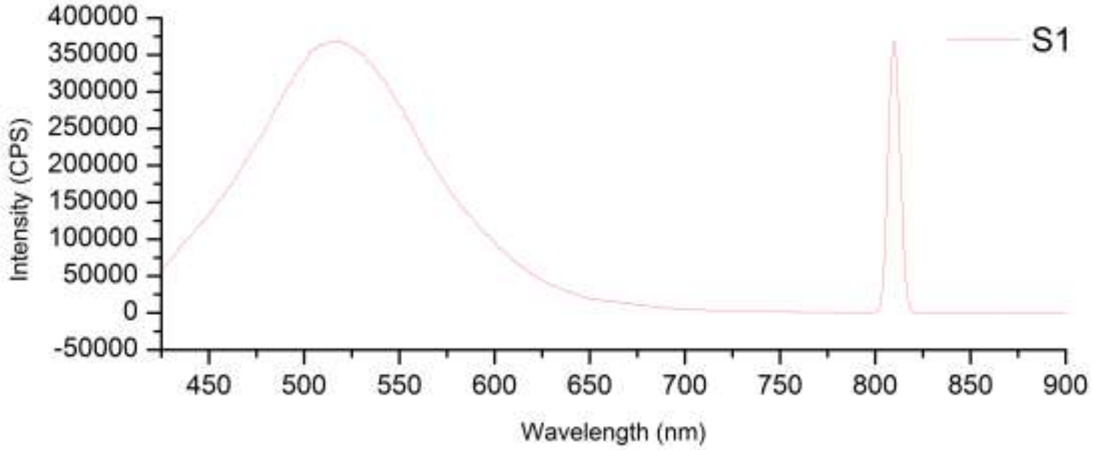

b) Phosphate buffer only, pH 7.5, Excitation 405 nm

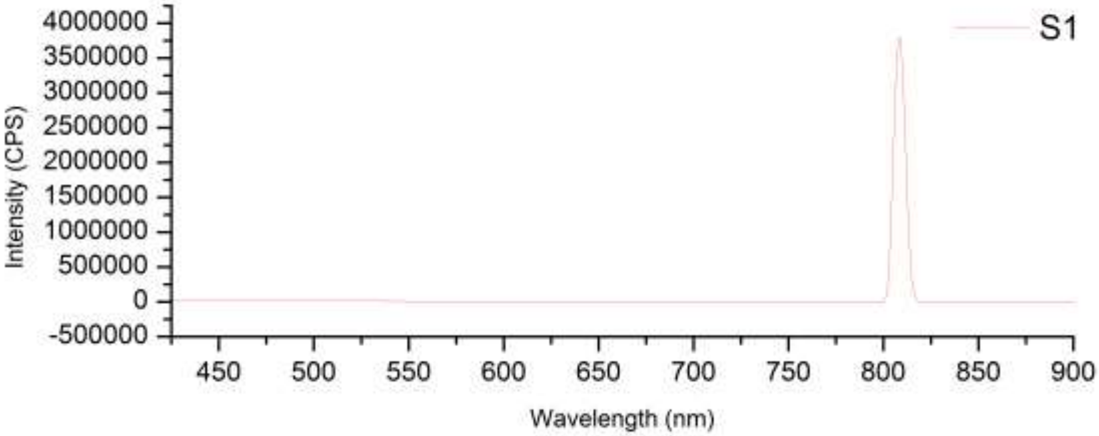

c) Phosphate buffer, pH 6.5, RM-581-Fluo (50 µM), Excitation 405 nm

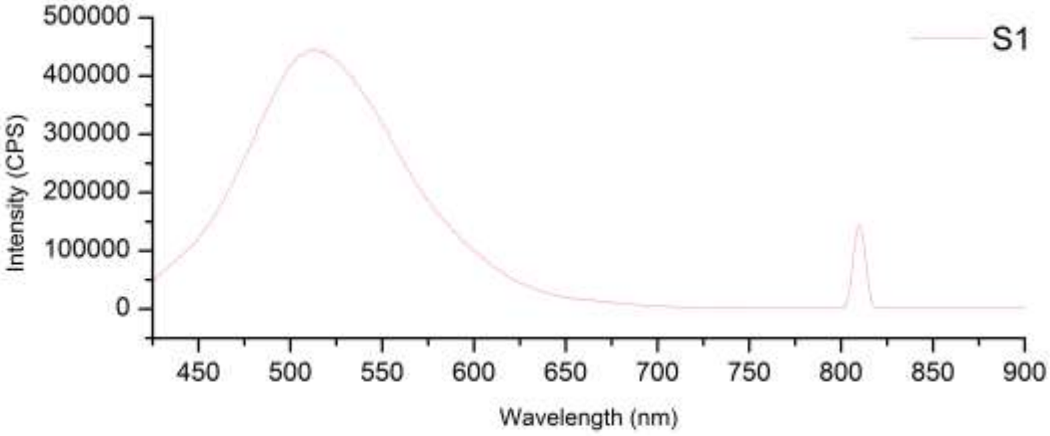

d) Phosphate buffer, pH 5.5, RM-581-Fluo (50 µM), Excitation 405 nm

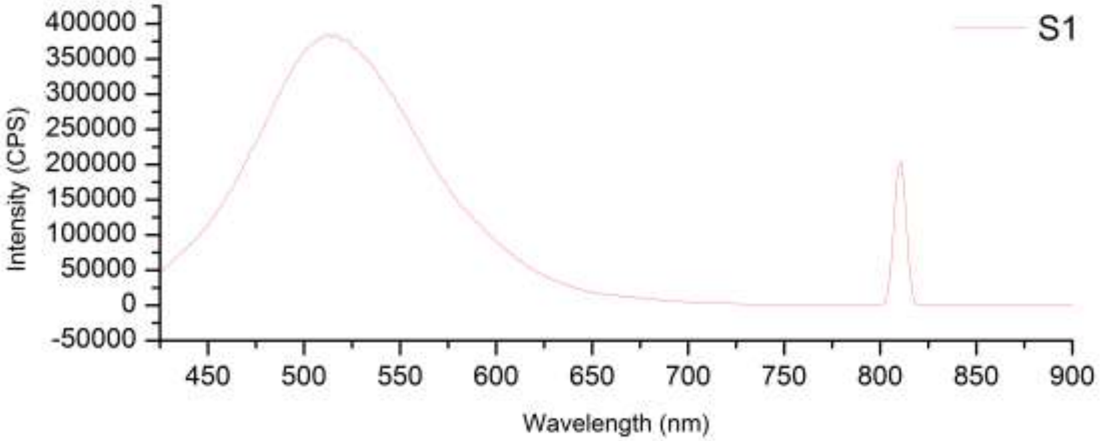

e) Phosphate buffer, pH 7.5, RM-581-Fluo (30 µM), Excitation 405 nm

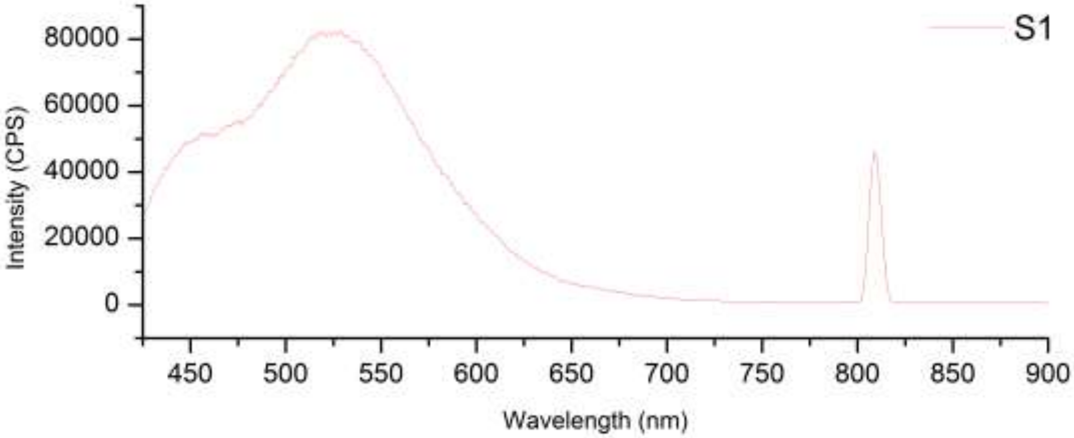

f) Phosphate buffer, pH 7.5, RM-581-Fluo (5 µM), Excitation 405 nm

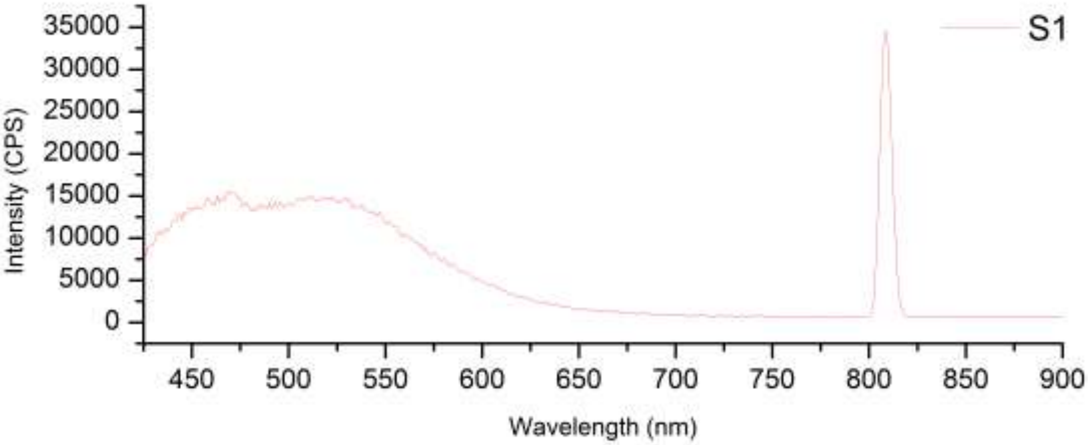

Figure S3. Three additional confocal imaging pictures from a second experiment with RM-581-Fluo in PC-3 cells. PC-3 cells were treated 2 h with 15 µM of RM-581-Fluo prior microscopy. (**A**) Cellular localization of RM-581-Fluo in blue (λex 405 nm, λem 460/50 nm); (**B**) Cellular localization of RM-581-Fluo in green (λex 405 nm, λem 525/50 nm); (**C**) Cellular localization of ER-Tracker in red (λex 561 nm, λem 593/40 nm); (**D**) Cellular localization of Cell-mask in cyan (λex 642 nm, λem 700/75 nm); (**E**) Merged images A, C and D showing the localization of RM-581-Fluo (in blue), ER tracker (in red) and Cell-mask (in cyan); (**F**) Merged images B, C and D showing the localization of RM-581-Fluo (in green), ER tracker (in red), Cell-mask (in cyan) and a co-localization in yellow of RM-581-Fluo and ER-Tracker. Scale bar: 10 µm.

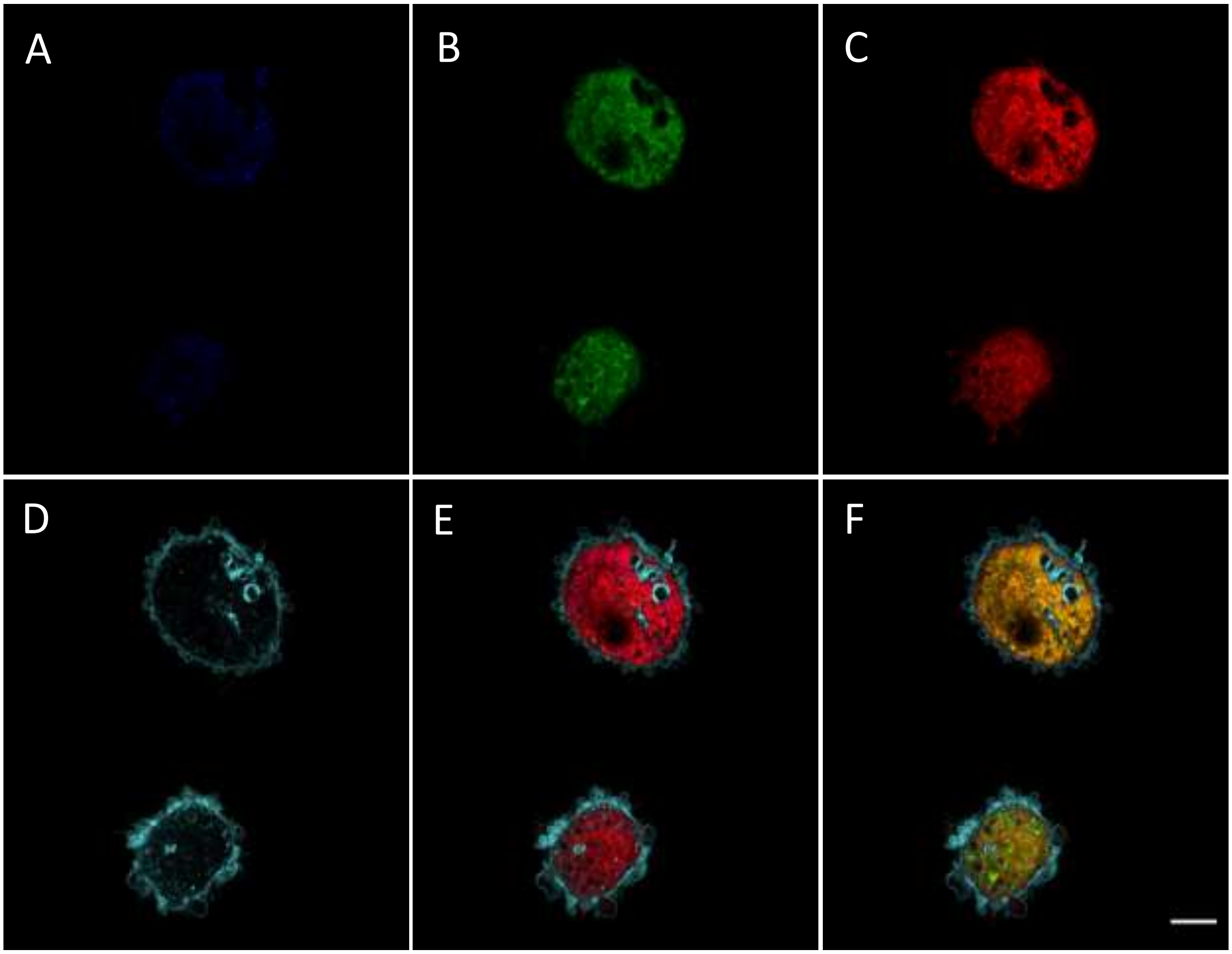

Figure S3 (follow)

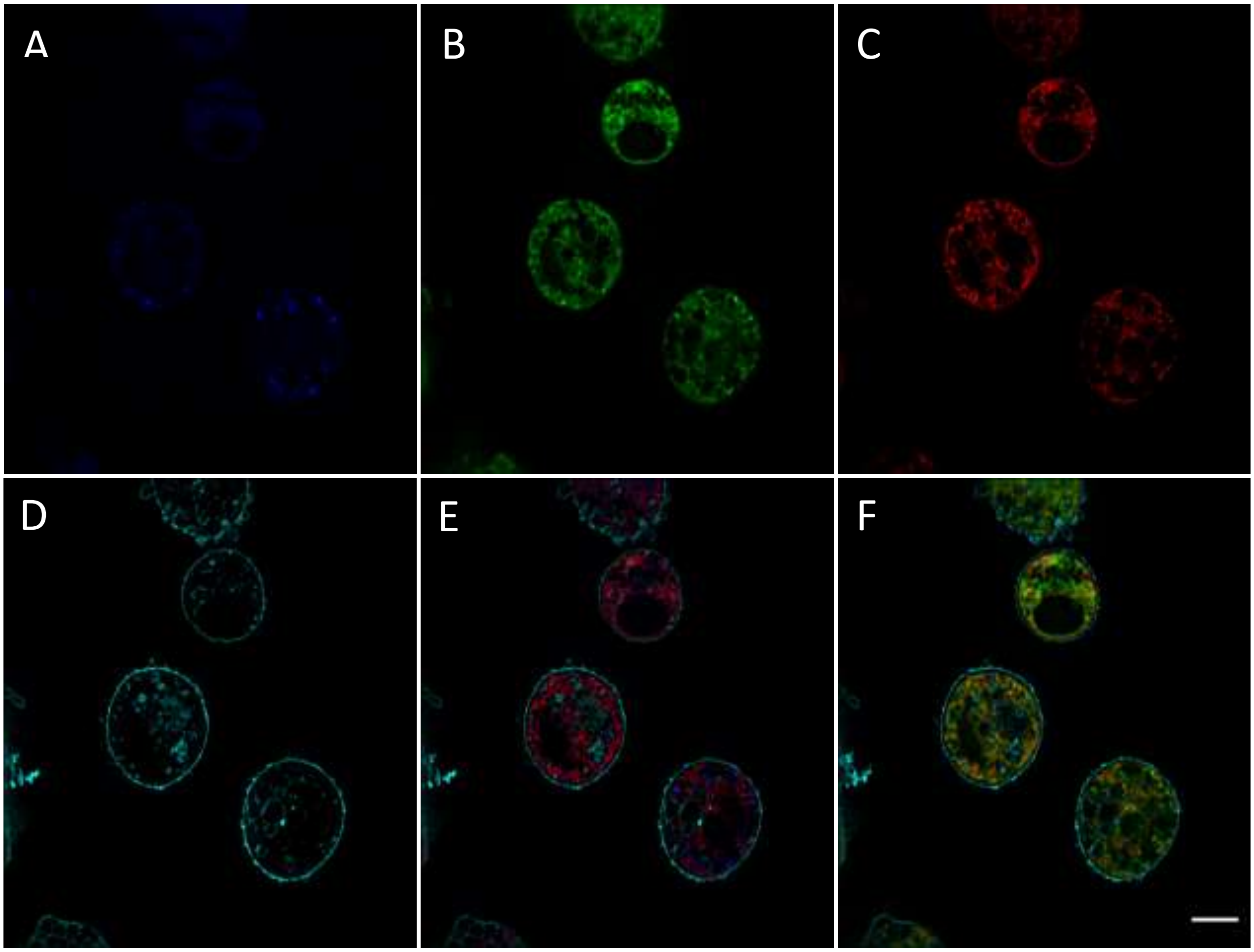

Figure S3 (follow)

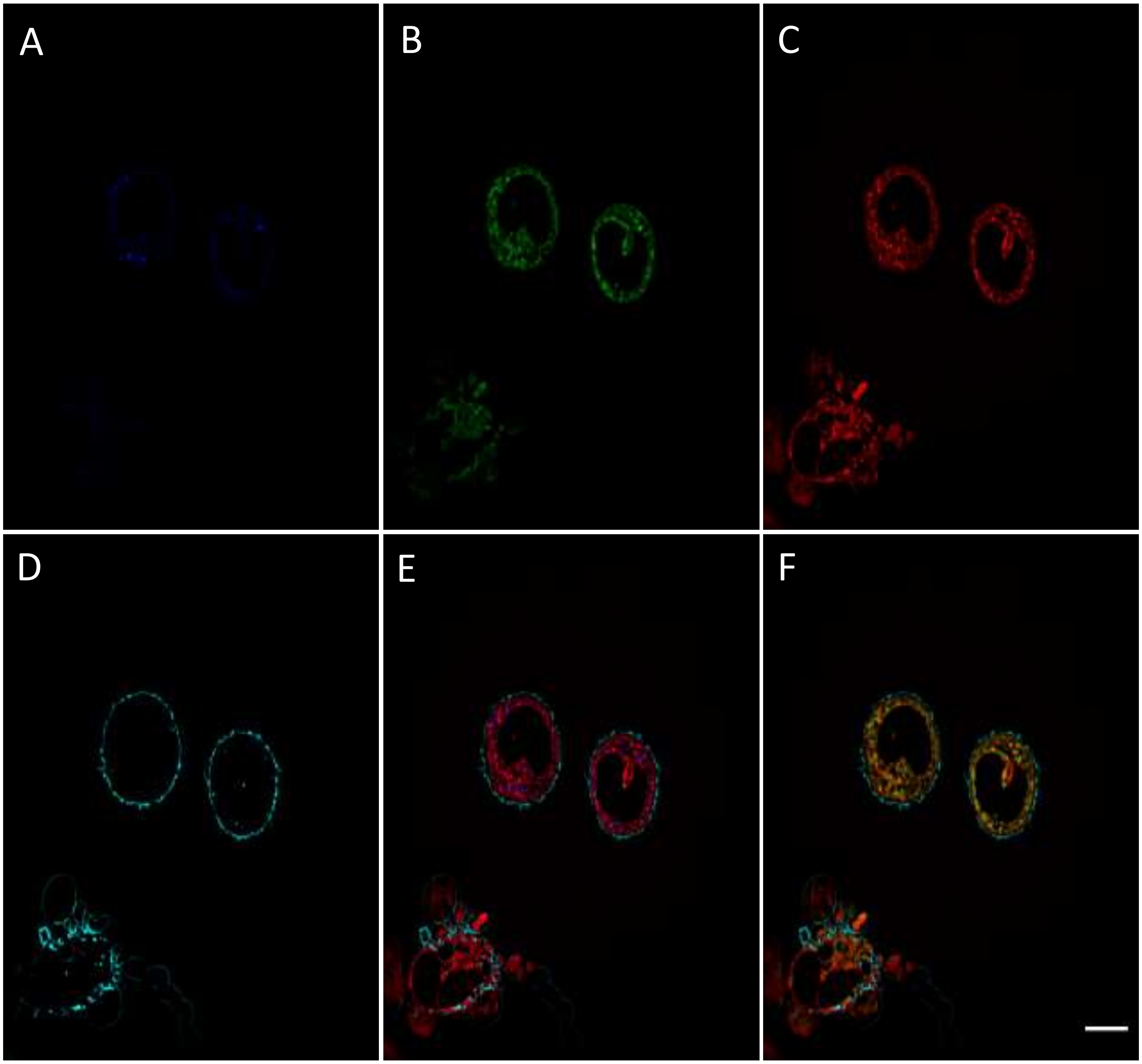

Figure S4. Cell proliferation and EC_{50} values after continuous exposure to RM-581 in PC-3 cells. PC-3 cells did not develop resistance when treated with 0.8 µM of RM-581. Cell proliferation and EC_{50} values after 0 (**A**), 12 (**B**), 49 (**C**) and 119 (**D**) days of treatment with RM-581. EC_{50} values are not significantly different.

A (EC_{50} = 1.2 ± 0.8 µM)

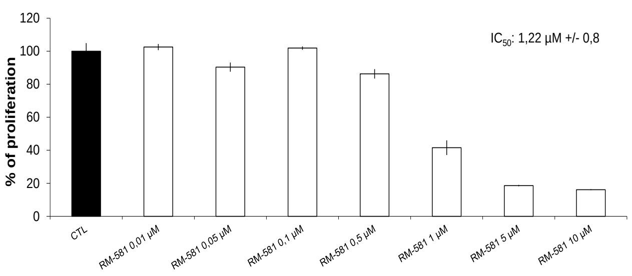

B (EC_{50} = 1.1 ± 0.5 µM)

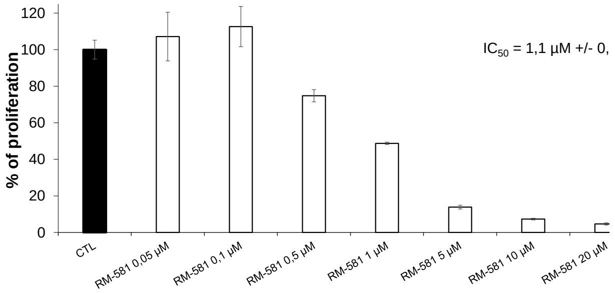

C (EC_{50} = 1.6 ± 0.6 µM)

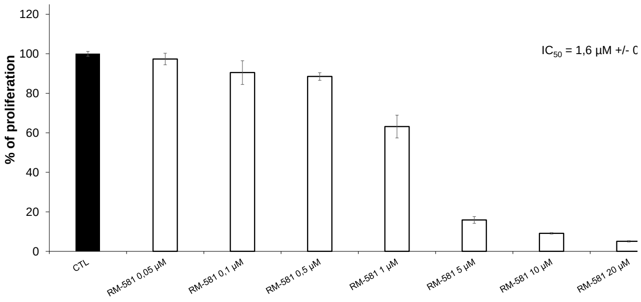

D (EC_{50} = 0.8 ± 0.4 µM)

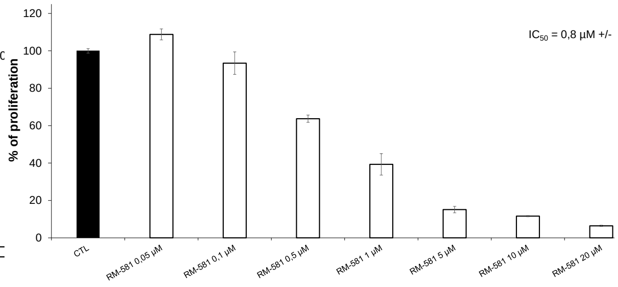

Supplement: Supplementary file 1 [file ijms-22-11181-s001.zip › ijms-1352886-supplementary.pdf]
